# Forecasting of infectious disease epidemics in sexual networks using attenuated renewal processes

**DOI:** 10.1371/journal.pcbi.1013987

**Published:** 2026-10-13

**Authors:** William D. Green, Christopher E. Overton, Thomas Ward, Ciara V. McCarthy, Sebastian Funk

**Affiliations:** 1 Centre for Mathematical Modelling of Infectious Diseases, London School of Hygiene and Tropical Medicine, London, United Kingdom; 2 Modelling Division, Analysis and Intelligence Assessment, Chief Data Officer Group, United Kingdom Health Security Agency, London, United Kingdom; 3 Department of Mathematical Sciences, University of Liverpool, Liverpool, United Kingdom; University of Sussex, UNITED KINGDOM OF GREAT BRITAIN AND NORTHERN IRELAND

## Abstract

Forecasting emerging infectious disease outbreaks is particularly challenging for sexual networks of men who have sex with men (MSM), where transmission can be shaped by heterogeneous and overdispersed contact structures, mixing assortativity, and both one-time and recurrent partnerships. Using stochastic epidemic simulations on synthetic MSM networks and empirical mpox case data from major cities, we systematically evaluated five classes of short-term forecasting models, from random walk approaches to semi-mechanistic attenuated renewal processes. Model performance was assessed using the continuous ranked probability score (CRPS). Across both simulated and empirical epidemics in heterogeneous networks, renewal models attenuated by a reverse-sigmoid curve, to account for early depletion of highly-connected nodes, consistently outperformed alternatives, achieving more balanced and reliable forecasts. Our findings suggest a practical approach for robust forecasting in highly overdispersed networks, where distinguishing network effects from other factors remains challenging and usually requires additional data.

## Author summary

Forecasting infectious disease outbreaks is particularly difficult in networks of men who have sex with men (MSM). These networks are structurally complex: contact rates are highly skewed, partnerships are shaped by degree- and role-based assortativity, and transmission can occur through both one-time and recurrent partnerships. Together, these features produce epidemic dynamics that are hard to predict with standard models, which typically assume contacts are distributed more evenly across a population.

A key consequence of this network structure is that highly connected individuals tend to be infected early, rapidly depleting the pool of susceptible people most likely to transmit. This causes the epidemic’s reproduction number to decline faster than models assuming homogeneous mixing would predict. Using stochastic epidemic simulations on synthetic MSM networks and empirical mpox case data from London, New York, San Francisco, Madrid, and Barcelona, we evaluated five classes of short-term forecasting model, including three novel attenuated renewal models. Across both simulated and empirical epidemics, renewal models in which the reproduction number decays along a reverse-sigmoid curve - explicitly accounting for early depletion of highly connected individuals - consistently outperformed alternatives. Our findings offer a practical approach to robust short-term epidemic forecasting in overdispersed sexual networks.

## Introduction

Men who have sex with men (MSM) represent an epidemiologically unique population whose social and behavioral characteristics confer a particular vulnerability to infectious disease outbreaks. The densely connected and highly over-dispersed nature of MSM sexual networks [1–3], in addition to the biological efficiency of transmission through anal intercourse [4] has contributed to epidemic emergence and endemic persistence of several pathogens in this population. This was seen most recently with the global mpox outbreak of 2022, when sustained transmission occurred almost exclusively within MSM communities across Europe and North America, outside of regions where the virus had previously been endemic [5]. Similar patterns have been observed with other pathogens that disproportionately affect MSM, including the long-standing HIV epidemic [6], recurrent syphilis outbreaks [7,8], the emergence of drug-resistant gonorrhea [9], and sexually transmitted enteric infections such as Shigella [10].

In light of this notable vulnerability, reliable epidemic forecasting in MSM populations is essential for effective public health responses. Accurate projections enable public health agencies to anticipate healthcare demand, allocate resources efficiently, and implement proportionate interventions to reduce morbidity and mortality [11].

Empirical studies have shown that MSM sexual networks are characterized by highly overdispersed degree distributions, in which a small fraction of individuals account for a large share of sexual partnerships. For example, simulated partnership distributions based on the UK National Survey of Sexual Attitudes and Lifestyles (Natsal) data in Endo *et al.* [1] implied that around 1% of MSM have more than 100 unique sexual partners over a year. MSM networks feature a greater degree of both one-time and concurrent partnerships, where the same contacts occur repeatedly over time [12,13], as well as diverse risk behaviors such as venue attendance, engagement in group sex, or participation in chemsex [14,15].

While first-order properties such as the degree distribution can be measured in behavioral surveys [16,17], higher-order structural characteristics, including degree assortativity [18,19], role assortativity [20] and spatial mixing are more difficult to quantify. Yet these properties could exert substantial influence on epidemic dynamics.

Forecasting is further complicated by the uncertainty surrounding key epidemiological parameters in the early stages of an outbreak. For emerging pathogens, or pathogens entering new risk networks, quantities such as the per-act transmission probability, directionality of transmission between subgroups, and the impact of behavioral interventions are often unknown [21]. Forecasting methods that rely on precisely specified network and transmission parameters may therefore produce inaccurate or unstable predictions when applied early in an outbreak. Addressing these challenges requires approaches that are robust to incomplete knowledge of network structure and adaptable to evolving epidemiological evidence.

Additional uncertainty comes from susceptible depletion which is amplified in heterogeneous networks where high-degree nodes can be infected, and thus removed from the susceptible population, disproportionately early [22,23]. As a consequence, one would expect the effective reproduction number to decline faster than would be predicted under homogeneous mixing. However, the magnitude and timing of this effect will depend on network properties such as degree distribution, assortativity and clustering, that are rarely known with precision during an emerging outbreak. This motivates forecasting approaches that can flexibly capture accelerated depletion without requiring detailed network specification.

Previous studies have not systematically evaluated the sensitivity of outbreak forecasts to uncertainty in network topology and early-outbreak transmission parameters, particularly in the context of high-risk MSM sexual networks. This gap limits our ability to design forecasting tools that are both operationally useful and resilient to the uncertainties inherent in emerging epidemics.

Here, we address this gap by developing a simulation framework to generate synthetic MSM sexual networks to simulate outbreak dynamics under a range of epidemiologically plausible scenarios. We apply a suite of flexible forecasting models - from statistical to semi-mechanistic approaches incorporating flexible attenuation of the reproduction number - to these simulated outbreaks to assess the robustness of their performance in highly overdispersed networks. We further assess forecast performance through a retrospective study on the 2022 Mpox outbreak in five cities: London, New York, San Francisco, Madrid and Barcelona.

## Methods

### Study design

Individual epidemics provide too few trajectories to reliably distinguish between forecasting approaches, and therefore do not offer sufficient statistical power to draw firm conclusions about comparative model performance. For this reason, our study combines both a synthetic and an empirical component. The synthetic component enables a controlled evaluation across many independently generated epidemic trajectories, while the empirical component evaluates performance on real-world outbreak data from London, New York, San Francisco, Madrid, and Barcelona. A schematic of the study is shown in Fig 1.

**Fig 1:**
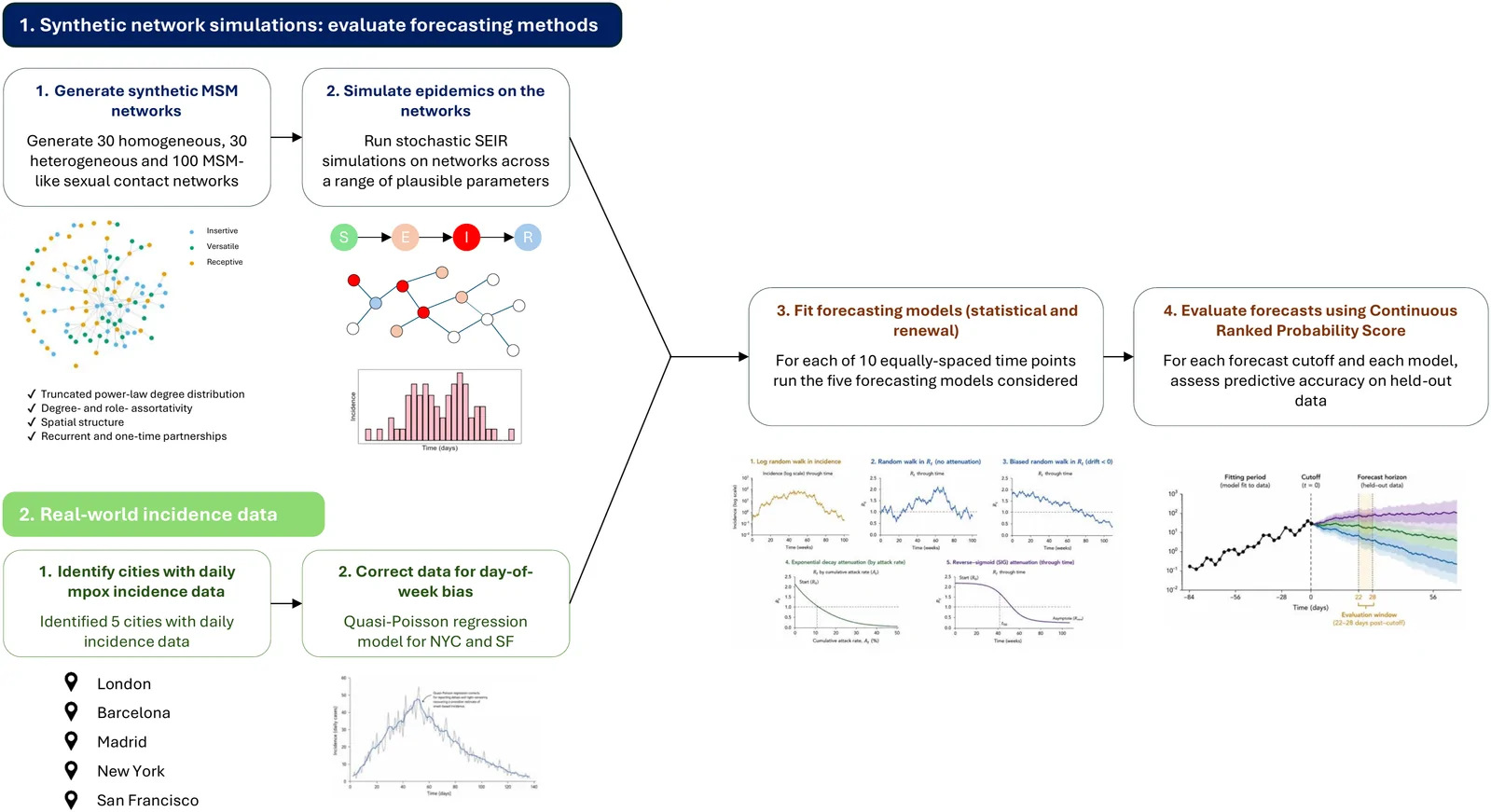
Schematic of the forecasting framework. Synthetic MSM sexual contact networks were generated and used to simulate stochastic SEIR epidemics across a range of epidemiologically plausible scenarios. Simulated incidence data, together with daily mpox incidence data from five cities, were used to fit alternative forecasting models. For New York and San Francisco, quasi-Poisson regression was applied to correct incidence reported by date of report for reporting-date biases. Forecasting models incorporating different assumptions about epidemic attenuation were fitted at multiple epidemic time points and evaluated using the Continuous Ranked Probability Score (CRPS) against incidence observed 22–28 days after the forecast cutoff.

In the synthetic analysis, we simulate epidemics across three classes of networks: (i) 30 simple regular networks in which every node has an identical degree (henceforth referred to as’homogeneous’ networks), (ii) 30 simple heterogeneous networks with degree distributions drawn from a truncated power-law, and (iii) 100 realistic MSM-like networks characterised by an overlapping one-time and recurrent partner structure. Epidemics are generated on each network instance and the five forecasting models are then fitted to the resulting epidemic curves to assess comparative performance under distinct structural assumptions.

In the empirical analysis, we apply the same modelling approaches to real-world epidemic curves in each of the five cities, enabling evaluation under realistic conditions with true behavioural, demographic, and reporting patterns. The code used to generate the analyses and figures in this study is publicly available at https://github.com/willgreen93/attenuated-renewal.

### Data

Data for the mpox outbreak in London was provided by the United Kingdom Health Security Agency (UKHSA) [24]. This gave us daily incidence by time of symptom onset, and so was considered agnostic to day-of-week effects in reporting. Data for Madrid and Barcelona was sourced from David García-García *et al.* [25]. This provided a time-series of daily incidence based on the date of onset, less a fixed incubation period of 7 days.

Data for San Francisco is taken from the publicly available surveillance dashboard by the San Francisco Department of Public Health (SFDPH) [26]. The dataset reports incident cases by date of report. Data for New York was sourced from the New York City Department of Health and Mental Hygiene (NYC DOHMC) [27], with cases reported by date of diagnosis. Both datasets are therefore potentially liable to day-of-week reporting artefacts arising from clinic operating hours and administrative processing. We applied a diagnostic check to each series by fitting a generalised linear model with quasi-Poisson errors and a log link (appropriate for overdispersed count data) regressing daily case counts on day of week. Quasi-Poisson errors accommodate the overdispersion typical of routine surveillance data, and the log link imposes a multiplicative structure whereby weekend suppression acts as a constant proportional scaling of true incidence regardless of epidemic magnitude. We apply this correction as a pre-processing step rather than adjusting within the models in order to keep the observation model identical across the five approaches and support comparison of the approach to forecasting latent dynamics. For New York City, day-of-week effects were substantial (S2 Fig, S2 Table). For San Francisco, a similar day-of-week correction was applied but with more limited impact on the resulting epi-curve (S2 Fig, S2 Table). We divided observed daily counts by the corresponding weekday-specific factors to yield an adjusted series preserving the underlying epidemic trend.

For all cities we only use data from the first wave of the outbreaks in 2022, with the forecasting model fit with a start point 7 days prior to the first case.

### Network generation

We developed a novel generative framework for synthetic MSM sexual contact networks that incorporates seven structural features empirically documented in this population: heavy-tailed degree distributions, in which a small fraction of individuals account for a disproportionate share of sexual partnerships [1]; a distinction between one-time and recurrent partnerships, which imply different contact frequencies and generation time distributions [12, 13]; sexual role structure and assortativity (insertive, receptive, versatile), which constrains partnership compatibility and influences transmission risk [4]; degree assortativity, governing whether high-degree nodes preferentially connect to one another [18, 19]; spatial mixing [28]; partnership duration, allowing for either one-time or recurrent interactions. The motivation for jointly incorporating these features was that their effects on network topology, and thus on epidemic dynamics, are non-trivial in combination. In particular, the interaction between degree overdispersion and assortativity determines how rapidly high-degree, high-risk nodes are depleted early in an outbreak, which is central to the forecasting problem studied here. Existing generative models for MSM contact networks typically incorporate one or two of these properties [1, 29]; combining all five within a single probabilistic edge-formation framework enabled a comparison of how well forecasting approaches perform across a range of network complexities.

The synthetic component of the analysis includes three classes of sexual contact networks, each reflecting a different level of structural realism. The *homogeneous* networks consist of 30 simulations in which all networks have the same degree and there is no role structure or assortativity. The homogeneous network was included as a theoretical comparator to isolate the effect of degree heterogeneity, not as a realistic representation of sexual contact patterns. The *heterogeneous* networks consist of 30 simulations with truncated power-law degree distributions but no recurrent partnerships or explicit role assortativity. Finally, the *realistic MSM-like* networks consist of 100 simulations parameterised using the seven structural properties described previously. The number of realisations differs across network classes to reflect their respective parameter space dimensionality (Table 1). Homogeneous and heterogeneous networks have fewer free parameters and serve primarily as benchmarks under simplified structural assumptions so 30 realisations are used to characterise model performance in these settings. MSM-like networks are parameterised by many more structural properties, motivating a larger number of realisations (n = 100) to adequately cover the higher-dimensional parameter space and ensure robust comparisons across network configurations.

**Table 1:** Parameter ranges sampled from the latin hypercube for each of the 30 homogeneous, 30 heterogeneous and 100 MSM-like networks. Homogeneous networks impose no assortativity or spatial structure; heterogeneous networks include degree heterogeneity but not assortative or role-based structure; MSM-like networks include full assortativity, role structure, and recurrent partnership dynamics. Parameters not applicable to a given network type are left blank. ^*a*^range estimated from [30], ^*b*^based on distribution of main and concurrent casual partnerships reported [31], ^*c*^approximate reasonable distribution taken from [32, 33].

Parameter	Symbol	Homogeneous	Heterogeneous	MSM-like
Network size	*N*	5,000 - 10,000	10,000 - 50,000	10,000 - 50,000
Transmission probability	*p*_trans_	0.2 - 0.5	0.2 - 0.5	0.2 - 0.5
Incubation period (days)	*T*_inc_	5 - 14	5 - 14	5 - 14
Infectious time (days)	*t*_inf_	15 - 45	15 - 45	15 - 45
Homogeneous degree	*k*	40 - 100		
Degree dist. parameter	α		1.6 - 2	1.6 - 2^*a*^
Degree upper bound	$k_{\max}$		100 - 500	100 - 500^*a*^
Daily recurrent contact prob.	*p*_rec_			0.05 - 0.2^*b*^
Assortativity kernel	$\zeta_{input}$			-0.5 - 1
Role assortativity parameter	$\rho_{input}$			-0.5 - 1
Spatial kernel scale	$\varepsilon_{input}$			0 - 3
Proportion versatile	$p_{vers}$			0.4 - 0.6^*c*^
Directionality	*d*			1 - 5

In the realistic MSM-like simulations, the contact structure is represented as two overlapping networks: a main (recurrent) partner network, where sexual activity occurs with probability *p*_rec_ per day, and a one-time partner network in which contacts occur only once over the simulation period. The one-time network was constructed first, with each edge in the one-time network assigned a single contact day drawn uniformly at random over the simulation period. The recurrent network was constructed second, with an edge between two nodes disallowed in the recurrent network if they were already connected in the one-time network.

Nodes were first assigned overall degrees according to the specified degree distribution, and edges were then formed probabilistically. The probability of an edge between nodes *i* and *j* was based on the product of four factors: the available degree of *j* ($d_{j}$), degree assortativity ($A_{ij}$), role compatibility ($R_{ij}$), and spatial proximity ($S_{ij}$) (equation 1). Edges were assigned iteratively: for each node *i*, a set of eligible partner nodes *j* > *i* was identified based on remaining degree capacity, role compatibility and spatial distance.

In both the one-time and the recurrent network, for each eligible partner, the edge formation probability was computed according to Equation 1, and a set of partners was sampled without replacement weighted by these probabilities. After each connection, the available degrees for both nodes were updated to ensure the final network matched the specified degree sequence.


$$\begin{array}{c} P(i↔j)=d_{j}A_{ij}R_{ij}S_{ij}/\sum_{l}d_{l}A_{il}R_{il}S_{il} \end{array}$$ (1)

The degree of node *j*, $d_{j}$ was sampled from a truncated power-law distribution:


$$\begin{array}{c} P(d_{j}=k)=\frac{k^{-\alpha }}{\sum_{m=1}^{k_{\max}}m^{-\alpha }},\;1\leq k\leq k_{\max}. \end{array}$$ (2)

Degree assortativity (see Fig 2) was described by a parameter ζ whereby in the case (ζ=0) there was no preferential mixing between nodes of a similar degree. Increasing ζ resulted in degree assortativity, where nodes connect more preferentially based on the relative difference in the overall number of partners, described by:


$$\begin{array}{c} A_{ij}=\exp\left(-\zeta \;\left|\log(d_{i})-\log(d_{j})\right|\right). \end{array}$$ (3)

**Fig 2:**
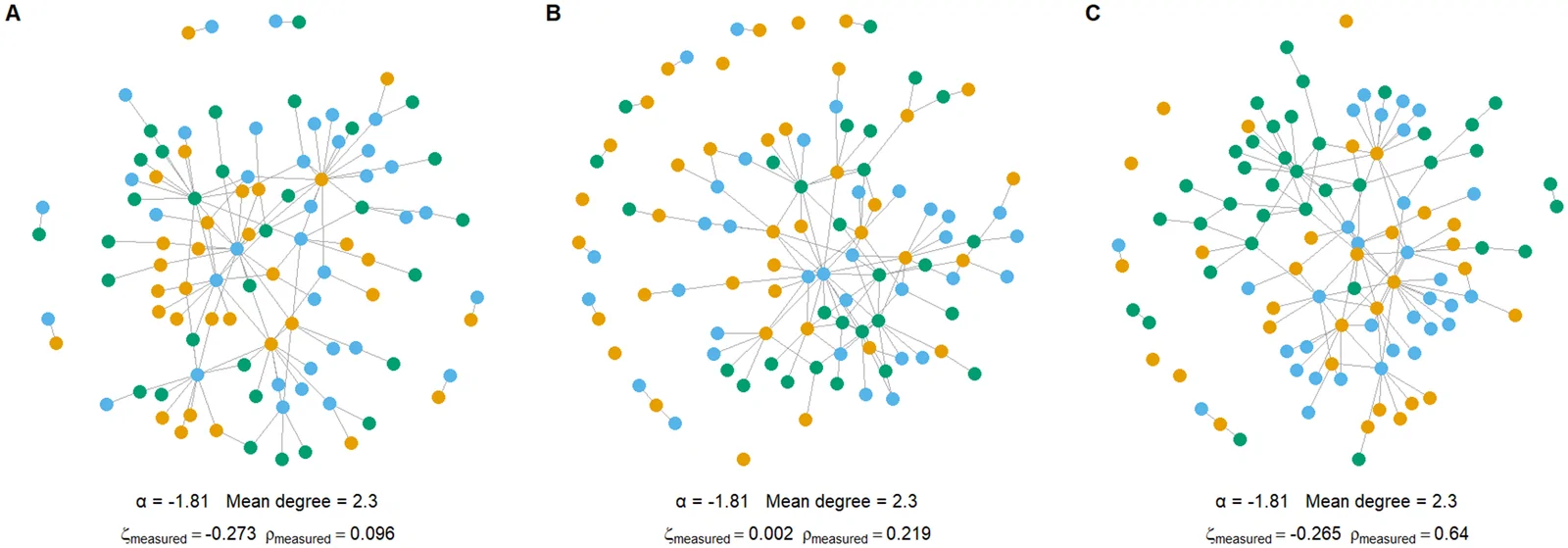
Examples of plausible networks with 100 nodes sharing the same power-law exponent (α=−1.81) and mean degree but with differing levels of degree assortativity (ζ) and role assortativity (ρ). Insertives are blue, receptives orange, versatiles green. **(A)** Base network with no preferential degree or role mixing; measured degree assortativity is negative as expected for truncated power-law degree distributions. **(B)** Network with increased degree assortativity ($\zeta_{\text{measured}}=0.002$), where high-degree nodes connect preferentially with other high-degree nodes. **(C)** Network with increased versatile assortativity ($\rho_{\text{measured}}=0.640$), where versatile nodes connect preferentially with other versatiles and receptive/insertive nodes connect preferentially with insertive/receptive nodes.

Spatial proximity was incorporated as a node-level attribute: each node was assigned coordinates drawn uniformly from the unit square, and the probability of edge formation between nodes *i* and *j* was modulated by an exponentially decaying spatial kernel parameterised by ε (equation 4). This reflects the empirical finding that sexual partnerships are more likely to form between geographically proximate individuals [28], thus the spatial parameter influences network topology during edge formation.


$$\begin{array}{c} S_{ij}=\exp\left(-\varepsilon \;\left\|(x_{i},y_{i}),(x_{j},y_{j})\right\|\right) \end{array}$$ (4)

Role assortativity (Fig 2) described the extent to which versatile nodes mix with versatile nodes, or, equivalently, the extent to which insertive/receptive partners seek out receptive/insertive partners respectively. This is composed of a matrix $R_{ij}$ (with columns and rows giving the mixing ratios of insertive, receptive and versatiles respectively) and a tuning parameter λ. In the extreme cases where λ is 2, versatiles mix exclusively with versatiles, and receptive/insertive nodes mix exclusively with insertive/receptive nodes respectively. When λ is −1, versatiles mix exclusively with receptive or insertive nodes, and do not engage in within-group mixing:


$$\begin{array}{c} R_{ij}=(\begin{array}{ccc} 0 & \frac{2}{3} & \frac{1}{3}\\ \frac{2}{3} & 0 & \frac{1}{3}\\ \frac{1}{3} & \frac{1}{3} & \frac{1}{3} \end{array})+\rho (\begin{array}{ccc} 0 & \frac{1}{6} & -\frac{1}{6}\\ \frac{1}{6} & 0 & -\frac{1}{6}\\ -\frac{1}{6} & -\frac{1}{6} & \frac{1}{3} \end{array}) \end{array}$$ (5)

To validate the generative framework, we measured key structural properties directly from the 100 generated MSM-like networks, including the realised power-law exponent, degree assortativity coefficient ($\zeta_{measured}$), versatile assortativity proportion ($\rho_{measured}$, defined as the proportion of edges incident to at least one versatile node that connect two versatile nodes), mean edge distance, and clustering coefficient. Each input parameter varied monotonically and strongly with its realised network property (Spearman correlation coefficient *r* > 0.90; S1 Fig), confirming that the generator provides control over the target structural features across a broad realised range.

Generated networks were not fully connected; across the 100 MSM-like realisations, the largest connected component contained a median of 86.2% of nodes (IQR: 83.3-88.2%, range: 74.1-93.3%). The second largest component never exceeded 1% of the size of the giant component, indicating that remaining nodes were largely isolated or in very small clusters rather than large competing sub-components. Two features of the epidemic simulation design (detailed below) ensured that epidemics propagated through the giant component in practice. First, the epidemic was seeded probabilistically in nodes with at least 10 connections, which are overwhelmingly located within the giant component given the heavy-tailed degree distribution. Second, simulations were rerun until a minimum of 500 cases were observed, effectively discarding any realisations in which the epidemic failed to propagate beyond a small isolated subcomponent.

### Epidemic simulation

Epidemics were simulated using a stochastic SEIR (Susceptible–Exposed–Infectious–Removed) framework on the generated networks, chosen for its generalisability to a broad range of infections. At each discrete time step, the probability that a susceptible individual *j* acquired infection from their infectious contacts was given by


$$\begin{array}{c} P\;\left(j_{S\to E}\right|t)=1-\prod_{i\in I(t)\cap {𝒩}_{j}}\left(1-\beta_{r_{i}r_{j}}\cdot c_{ij}(t)\right) \end{array}$$ (6)

where i∈I(t) denotes the set of infectious individuals at time *t*, $\beta_{r_{i}r_{j}}$ is the per-act transmission probabili*t*y determined by sexual role and directionality, and ${𝒩}_{j}$ represents the neighbourhood of *j*. The contact indicator $c_{ij}(t)\in \{0,1\}$ equals 1 if nodes *i* and *j* had sexual contact at time *t* and 0 otherwise. For recurrent partnerships, contact at each time step occurs with probability *p*_rec_; for one-time partnerships, contact occurs only on the single day assigned *t*o that edge during network construction.

The role-stratified transmission matrix $\beta_{r_{i}r_{j}}$ was constructed from a baseline transmission probability *p*_trans_ and a directionality parameter d≥1, which captures the elevated per-act transmission risk associated with the receptive role:


$$\begin{array}{c} \beta_{r_{i}r_{j}}=(\begin{array}{ccc} 0 & \frac{2p_{\text{trans}}d}{d+1} & \frac{2p_{\text{trans}}d}{d+1}\\ \frac{2p_{\text{trans}}}{d+1} & 0 & \frac{2p_{\text{trans}}}{d+1}\\ \frac{2p_{\text{trans}}}{d+1} & \frac{2p_{\text{trans}}d}{d+1} & p_{\text{trans}} \end{array})\begin{array}{c} \leftarrow \text{rec}\\ \leftarrow \text{ins}\\ \leftarrow \text{ver} \end{array} \end{array}$$

where rows index the infectee role and columns the infector role (rec = receptive, ins = insertive, ver = versatile). The parameterisation ensures that the mean of the insertive-to-receptive and receptive-to-insertive transmission probabilities equals *p*_trans_ for all values of *d*, so that *p*_trans_ retains its interpretation as an average per-contact transmission probability. When *d* = 1 all non-zero entries reduce to *p*_trans_, recovering a role-agnostic transmission model.

In all networks the epidemic was seeded with a single infection. In the heterogeneous and realistic MSM-networks, the index case was chosen probabilistically among individuals with more than ten edges with a probability proportional to the number of edges the individual has. Individuals who become infected entered the exposed compartment, *E* and remained there for a fixed incubation period before progressing to the infectious state, *I*. Infectious individuals remain infectious for a period $t_{inf}$, after which they transitioned to the removed state, *R*. The allowed parameter ranges for the network generation and epidemic simulation are given in Table 1.

### Forecasting approaches

We consider five different approaches for the forecast of daily incidence, *I*(*t*), spanning from purely statistical to attenuated renewal processes. This includes one statistical model, a model based on the renewal equation, and three extensions of this model where the reproduction number becomes attenuated over time.

Renewal process models provide a natural framework for short-term epidemic forecasting by expressing current incidence *I*(*t*) as a weighted sum of past incidence, with weights given by the generation time distribution, ω(τ) [34]:


$$\begin{array}{c} I(t)=R_{t}\sum_{\tau }I(t-\tau )\;\omega (\tau ), \end{array}$$ (7)

The effective reproduction number $R_{t}$ summarises current transmission intensity: values above one indicate epidemic growth, values below one indicate decline. Forecasting then reduces to specifying how $R_{t}$ is expected to evolve beyond the observation window: different assumptions about this evolution define the model comparison in this study.

The log-random walk (LRW) [35] serves as a purely statistical baseline, making no mechanistic assumptions about transmission and instead extrapolating recent incidence trends:


$$\begin{array}{c} \log(I_{t})\sim 𝒩(log(I_{t-1}),\sigma^{2}) \end{array}$$ (8)

For the renewal models with susceptible depletion, the daily incidence is generated as a function of the reproduction number *R*(*t*); the historic incidence *I*(*t*), the population size *N* and the generation time distribution ω(τ) by equation 9.


$$\begin{array}{c} I(t)=R(t)\left(1-\frac{\sum_{m<t}I(m)}{N}\right)\sum_{\tau }I(t-\tau )\;\omega (\tau ), \end{array}$$ (9)

where the term $\left(1-\frac{\sum_{m<t}I(m)}{N}\right)$ accounts for susceptible depletion under the assumption of homogeneous mixing.

The non-attenuated renewal equation model was based on a random walk in R, with a step-size of 3 days (*RRAND*) [36]:


$$\begin{array}{c} \log(R_{t})\sim 𝒩(log(R_{t-1}),\sigma^{2}) \end{array}$$ (10)

We further use a range of models where the reproduction number is attenuated over time in order to capture susceptible depletion in the network beyond what would be expected under homogeneous mixing. The models considered were as follows.

Biased random walk in *R*, also using a step size of 3 days [37] (*RBIAS*). RBIAS extends the random walk model (RRAND) by introducing a systematic downward drift δ, representing a natural augmentation in which $R_{t}$ retains the flexibility of a random walk but is subject to a constant pull towards lower values over time [37]. This drift acts as a simple, minimally-parameterised attenuation mechanism: without imposing any functional form on the trajectory of decline, it encodes the prior expectation that transmission intensity tends to decrease as an epidemic progresses, whether through susceptible depletion, behavioural change, or public health intervention.


$$\begin{array}{c} \log(R_{t})\sim 𝒩(log(R_{t-1})-\delta ,\sigma^{2}) \end{array}$$ (11)

A reverse-sigmoidal decay in R as a function of time (*SIGT*). The reverse-sigmoid functional form is motivated by the two-network structure of MSM sexual contact networks. Early in an epidemic, transmission is driven primarily by the one-time partner network, where high edge overdispersion results in rapid infection of high-degree nodes and a correspondingly high effective reproduction number. As these highly-connected individuals are depleted from the susceptible population, transmission increasingly reflects the recurrent partner network, where connectivity is lower and more homogeneous. The sigmoid was chosen as it captures a smooth transition from the high effective reproduction number characteristic of the early epidemic, driven by the one-time partner network, to the lower reproduction number of the later epidemic, dominated by recurrent partnerships, while yielding directly interpretable parameters. Other monotonically decreasing smooth functional forms, such as the Gompertz decay or generalised logistic, could in principle be considered but are left for future work.


$$\begin{array}{c} R_{t}=R_{\text{floor}}+\frac{R_{0}-R_{\text{floor}}}{1+e^{-\delta (T-t)}} \end{array}$$ (12)

Exponential decay in *R* as a function of attack rate (*EXPI*). Unlike the SIGT model, where attenuation is indexed by time, EXPI links the decline in *R* over and above homogeneous susceptible depletion also directly to the cumulative proportion of the population infected ($A_{t}$). This is motivated by the observation that highly-connected nodes are disproportionately likely to be infected at very low attack rates - even the earliest infections are concentrated among high-degree individuals - meaning high-connectedness depletion begins almost immediately when treated as a function of attack rate rather than calendar time. An exponential functional form, which declines monotonically from $A_{t}=0$ without an inflection point, is therefore more appropriate than a sigmoid when attenuation is indexed by attack rate rather than time. The inclusion of a floor parameter *R*_floor_ serves the same purpose as in the SIG model, capturing the residual transmission sustained by recurrent partnerships once one-time contact susceptibles are largely exhausted. EXPI therefore serves as a useful comparator to SIGT:


$$\begin{array}{c} R_{t}=R_{floor}+(R_{0}-R_{floor})\cdot e^{-\delta \cdot A_{t}},\;A_{t}=\frac{1}{N}\sum_{s=1}^{t-1}I_{s} \end{array}$$ (13)

For all models we use a Negative Binomial observation model for reported incidence:


$$\begin{array}{c} I_{\text{obs}}(t)\sim \text{NegativeBinomial}(I_{\text{model}}(t),\phi ), \end{array}$$ (14)

where ϕ is an overdispersion parameter.

Details of the model parameters and their prior distributions are provided in the supplementary material. (Table 1).

Prior distributions were chosen to be weakly informative, centred on epidemiologically plausible values while allowing the likelihood to dominate inference where data are sufficient (S1 Table). The prior on *R*_0_ was log-normal with median 10 and a 95% interval spanning 4.6 - 21.9, allowing for a broad range of early exponential growth. The overdispersion parameter ϕ is centred at 30, but again this was weakly informative with 95% bounds of 11.3 and 79.9, suggesting moderate overdispersion. Priors on attenuation parameters were chosen to permit a broad range of epidemic trajectories, from rapid to gradual decline. Shared parameters (*R*_0_, *I*(0), ϕ) use identical priors across all renewal models, ensuring that performance differences reflect model structure rather than prior specification.

### Fitting approach and evaluation

For the empirical analysis, the generation time distribution was fixed at τ~𝒰(7,21) days, assuming a 7-day incubation period [38] and 14-day infectious period [39], with transmission equally likely across the infectious period. MSM population sizes for each city were fixed from external estimates (S1 Table). In the synthetic analysis, both the network size *N* and generation time distribution ω(τ) were assumed to be known exactly, taken directly from the simulation parameters used to generate each epidemic. A complete list of fixed inputs and estimated parameters, presented separately for the synthetic and empirical analyses, is provided in the supplementary material (S1 Table).

For each simulation, forecasts were generated at ten equally spaced time points spanning the period from when 5% to 95% of all infections had occurred, corresponding to a median inter-forecast interval of 8.5 days (95% interval across simulations: 4.1-16.8 days). This spacing ensured that forecast origins were distributed across the full epidemic trajectory - encompassing growth, peak, and decline - rather than concentrated in any single phase. Each model predicted incidence 28 days into the future from each forecast cutoff, a horizon chosen to reflect the timescale over which public health authorities must plan resource allocation and intervention responses, and consistent with established infectious disease forecasting frameworks [40, 41]. For each forecast, the predicted and observed daily incidence were summed to weekly incidence, and the CRPS was computed on this weekly total. Model performance was assessed based on predicted incidence during days 22–28 post-cutoff (the final week of the horizon) rather than the full window. Near-term predictions (days 1–7) tend to be accurate across all models as they extrapolate from data close to the forecast cutoff, making early-horizon performance uninformative for model discrimination; longer-horizon accuracy is the more operationally relevant quantity, where decisions must be made well in advance of projected peaks or troughs. For completeness, mean CRPS decomposed by individual forecast week (days 1–7, 8–14, 15–21) is provided in the supplementary material (S3-S5 Tables).

Models were fit using Hamiltonian Monte Carlo (HMC) as implemented in the probabilistic programming language Stan [42]. For each simulation we initially ran four chains with 500 warm-up steps and 500 iterations. The number of sampling iterations was increased as needed until all parameters achieved convergence ($\hat{R}<1.05$). Divergent transitions were addressed by incrementally increasing adapt_delta. For a small number of edge-cases (0.2% of the combinations of model, epidemic-phase, and simulation number), divergent iterations or high $\hat{R}$ were not fixed with increasing adapt_delta or iteration count, so these were discounted from the analysis. HMC uses gradient-based methods to efficiently explore high-dimensional parameter spaces, typically achieving faster convergence and better mixing than traditional random-walk Markov Chain Monte Carlo (MCMC) algorithms.

Forecast accuracy was evaluated using the Continuous Ranked Probability Score (CRPS) as implemented in the scoringutils package [43]. For a predictive cumulative distribution function (CDF) *F* and an observed outcome *y*, the CRPS is defined as:


$$\begin{array}{c} CRPS(F,y)=\int_{-\infty }^{\infty }(F(z)-H(z-y){)}^{2}\;dz, \end{array}$$ (15)

where *H* is the Heaviside step function. Intuitively, the CRPS integrates the squared distance between the forecast CDF and the empirical CDF at the observed value. The CRPS rewards forecast distributions that place high probability mass close to the observed value: a sharp distribution well-centred on the observation scores well, while a distribution that is poorly centred, unnecessarily wide, or both is penalised. The CRPS therefore jointly assesses calibration (whether observations fall in plausible regions of the forecast distribution), and sharpness (whether the distribution is concentrated).

The CRPS can be decomposed into overprediction, underprediction, and dispersion terms, providing insight into whether model errors arise from systematic bias (forecasts being consistently too high or too low) or from insufficiently calibrated forecast uncertainty (too narrow or too wide predictive intervals).

We computed the Continuous Ranked Probability Score (CRPS) on both the linear and logarithmic scales as recommended in [44]. The linear scale facilitates evaluation of absolute features such as peak incidence or peak demand, while the logarithmic scale provides a measure of relative error, allowing more sensitive assessment of model performance across different epidemic magnitudes.

## Results

### Simulation studies on synthetic networks

To assess model performance under controlled conditions, we evaluated forecast accuracy across types of network epidemic with parameter combinations drawn by latin hypercube sampling. Model performance was assessed using mean Continuous Ranked Probability Scores (CRPS) on both linear and log-transformed incidence scales. The mean CRPS across each setting, for forecast period from day 22–28, is shown in Table 2. We show the tables for forecast periods 1–7, 8–14 and 15–22 in the Supplementary Material (S3-S5 Tables).

**Table 2:** Forecasting performance for days 22 to 28 across epidemic models. Values represent the mean Continuous Ranked Probability Score (CRPS) for each model across five real-world cities: Barcelona (BCN), London (LON), Madrid (MAD), New York City (NYC), and San Francisco (SF) and three simulated network types: homogeneous, heterogeneous, and MSM-like. Models considered are log random walk (LRW); a renewal model with random walk in $R_{t}$ (RRAND); a renewal model with a biased random walk in $R_{t}$ (RBIAS); a renewal model with exponential attenuation of $R_{t}$ with attack rate to a $R_{floor}$ (EXPI); and a renewal model with sigmoidal attenuation of $R_{t}$ through calendar time (SIGT). Lower CRPS values indicate better forecast performance. For each column, the best-performing model within each metric is shown in bold. Note that network size differs by class so CRPS can not be compared across columns.

Model	BCN	LON	MAD	NYC	SF	Homogeneous	Heterogeneous	MSM-like
**Linear CRPS**								
LRW	119	66	88	303	42	429	116	294
RRAND	970	381	474	1220	248	**86**	370	910
RBIAS	158	48	57	295	20	249	123	352
EXPI	137	439	265	970	277	459	87	156
SIGT	**83**	**28**	**56**	**180**	**17**	400	**62**	**125**
**Log CRPS**								
LRW	0.49	0.54	0.38	0.61	0.70	0.67	0.80	0.47
RRAND	1.57	1.11	0.89	1.02	1.19	**0.13**	0.77	0.67
RBIAS	0.47	0.34	**0.30**	0.61	0.35	0.40	0.39	0.34
EXPI	0.36	1.05	0.78	0.88	0.93	0.74	0.60	0.25
SIGT	**0.30**	**0.23**	0.37	**0.30**	**0.32**	0.64	**0.37**	**0.23**

In the homogeneous setting, the standard renewal model *RRAND* provided the most accurate forecasts. It achieved a mean CRPS of 86 on the linear scale and 0.13 on the log scale, outperforming the next best model (RBIAS) by 65% and 68% respectively. These findings align with expectations that classical renewal models perform well under assumptions of homogeneous mixing.

However, in the heterogeneous network setting, where transmission was concentrated among highly connected individuals, the performance of *RRAND* deteriorated sharply. It became the worst-performing model with a linear CRPS of 370 and log CRPS of 0.77. In contrast, the SIG model emerged as the most accurate across both the linear and log scales. This reversal in performance highlights the limitations of unconstrained renewal models in the presence of strong contact heterogeneity.

The SIG model also achieved the best performance in the realistic MSM network simulations. Here, the *SIG* model outperformed all alternatives. On the log scale it performed similarly to EXPI, but it achieved a 20% better performance on the linear scale. SIGT and EXPI outperformed all other models considered quite considerably.

In the MSM-like networks, an error decomposition by epidemic phase demonstrates the random walk model *LRW* exhibiting the expected temporal bias — under-predicting early and overpredicting late (Fig 3). The *RRAND* model overpredicted across all phases, which is expected given *R* starts higher in overdispersed networks as the epidemic progresses through highly-connected nodes. EXPI and SIGT both show a tendency to under-predict early in the epidemic (phases 1–3). When cumulative incidence is low, the data are weakly informative about the attenuation rate δ, so the posterior remains close to the prior. This may induce premature attenuation of $R_{t}$ (through δ in EXPI and the midpoint *T* in SIGT). We did not assess sensitivity to these priors. In later epidemic phases, this pattern reverses to a slight overprediction, suggesting that in both models attenuation lags behind the true structural decline in $R_{t}$.

**Fig 3:**
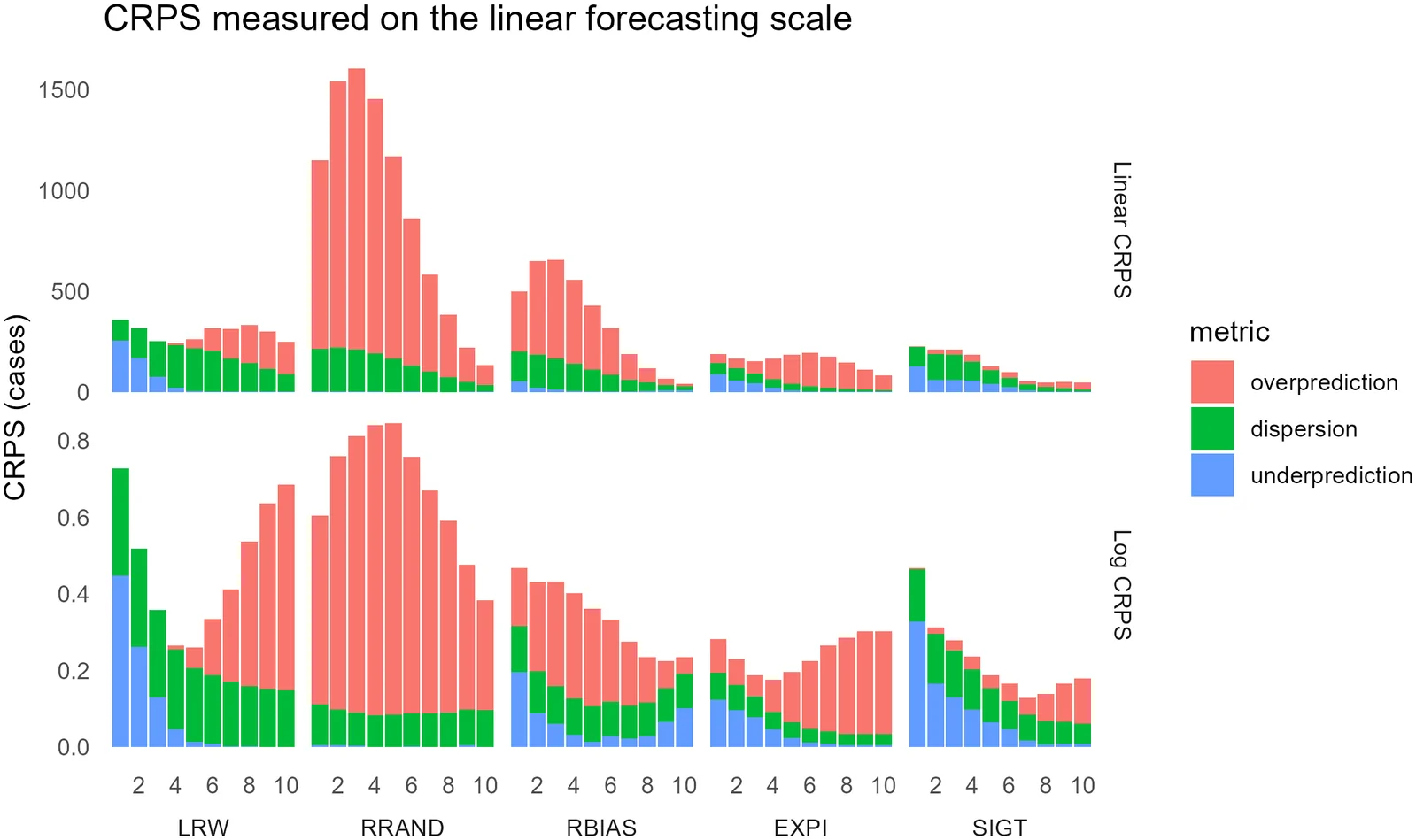
Decomposition of the Continuous Ranked Probability Score (CRPS) for the MSM-like networks into overprediction (red; forecast distribution places excess mass above the observation), dispersion (green; penalty for forecast spread, reflecting uncertainty in the predictive distribution), and underprediction (blue; forecast distribution places excess mass below the observation) for MSM-like network simulations (*n* = 100). Results are shown separately for the linear scale (top; y-axis in units of cases) and log scale (bottom; y-axis in units of log cases). The x-axis denotes epidemic phase, where earlier phases correspond to forecasts made during epidemic growth and later phases to forecasts made near or after the epidemic peak. A bar dominated simultaneously by large overprediction and underprediction components indicates a model that is highly variable across simulations - alternately projecting too high and too low - without sufficiently wide forecast intervals to capture this variation. For example, RRAND shows consistently large overprediction on the linear scale, particularly at early epidemic phases, reflecting the tendency of an unconstrained random walk to project continued exponential growth when the true epidemic is declining. By contrast, SIGT produces small bars across most phases, with residual error attributable primarily to dispersion, indicating well-centred forecasts with appropriately calibrated uncertainty rather than systematic directional bias.

Examples of the modelled case reproduction number trajectory against the true case reproduction number trajectories using the sigmoid-attenuated function are shown in Fig 4. The fitted trajectories capture the characteristic sigmoidal decline in $R_{t}$ observed across simulations, reflecting the temporal delay between epidemic onset and the preferential depletion of high-degree nodes that drives transmission slowdown in overdispersed networks. The attenuation midpoint *T* allows the model to flexibly encode when this hub-depletion effect transitions from a background process to the dominant driver of $R_{t}$ decline, accommodating the variation in network structure across simulations.

**Fig 4:**
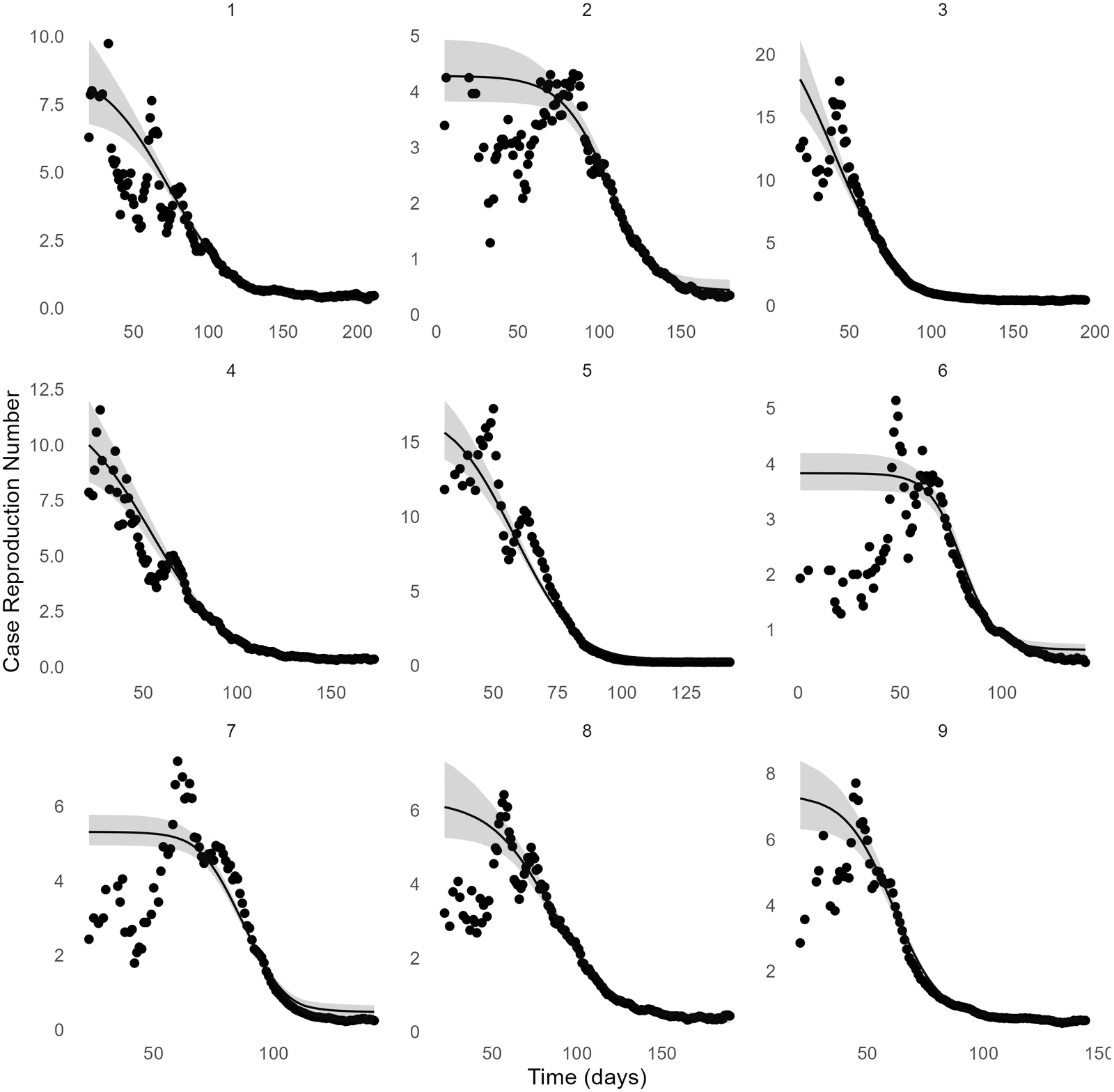
Comparison of daily case reproduction number to fitted trajectory using the SIG model for an example subset of epidemic simulations. Points show case reproduction number in the simulation, line and ribbon show the modelled trajectory with 95% CrI accounting for reverse sigmoid depletion and build-up of population immunity.

### Application to real-world epidemics

To evaluate model performance in real settings, each forecasting approach was applied to epidemic data from five major cities: London, New York City, San Francisco, Madrid, and Barcelona. The *SIG* model again demonstrated consistently strong performance, particularly on the linear scale, where it achieved the best fit in all five cities. On the log scale, it was the top performer in four cities and ranked second in Madrid (Table 2).

The city-level CRPS decomposition (Fig 5) reveals a consistent pattern of overprediction for RRAND and EXPI across all cities, concentrated in early epidemic phases (1–4) when epidemics are before their peak. This reflects the failure of unconstrained or slowly attenuating models to anticipate the structural slowdown in transmission: both models project continued exponential growth during the epidemic peak, generating forecasts that substantially exceed observed incidence. The magnitude of this overprediction was particularly severe in New York City, where RRAND reached a mean linear CRPS in excess of 4,000 cases at peak phases. By contrast, RBIAS and SIGT produced small, dispersion-dominated error bars across all cities and phases, indicating well-calibrated forecasts. The average rank of forecasting models across cities and epidemic phases is shown in S3 Fig.

**Fig 5:**
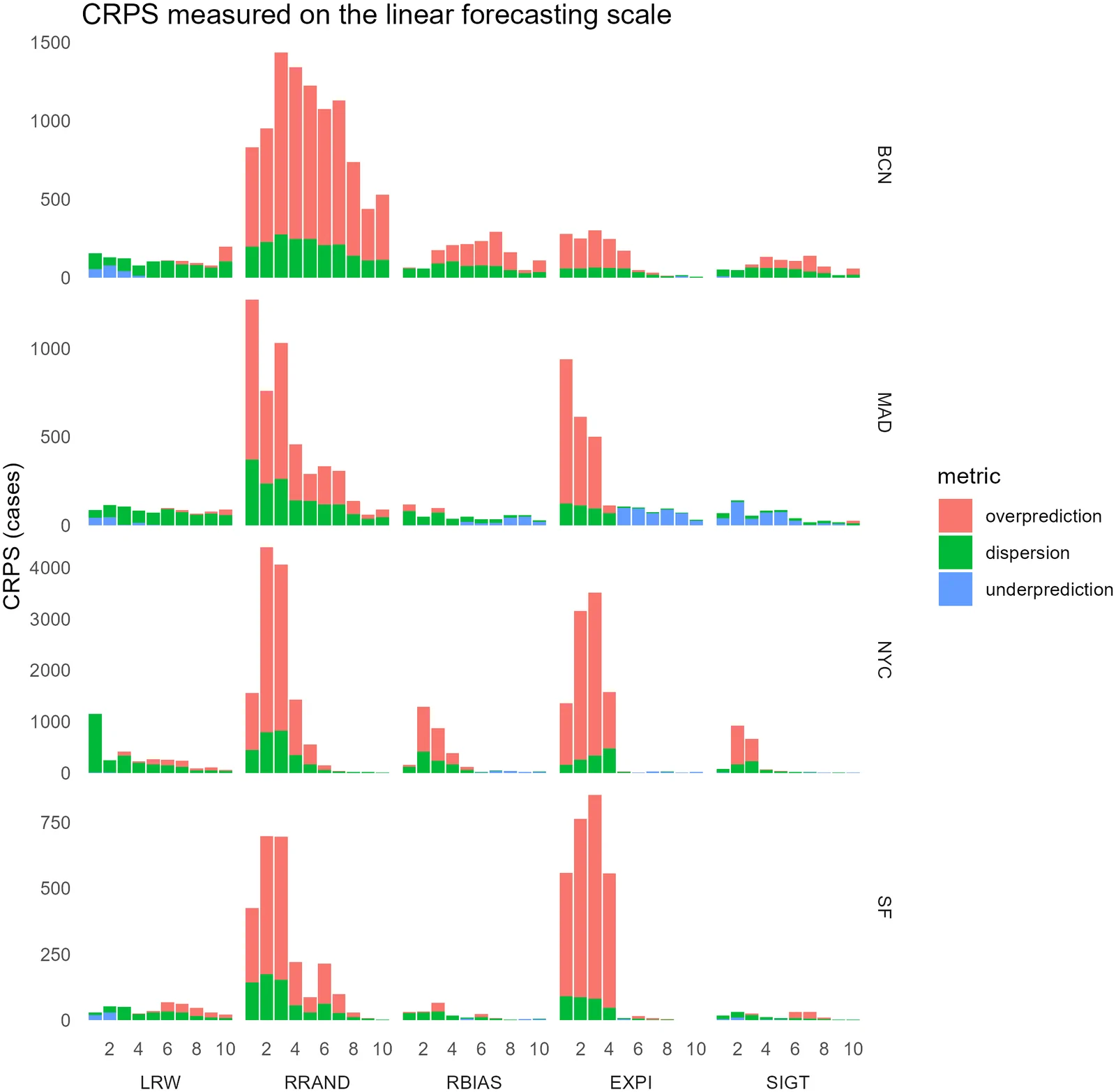
Decomposition of forecast error by epidemic phase and city. Continuous Ranked Probability Score (CRPS) across epidemic phases (x-axis) for Barcelona (BCN), Madrid (MAD), New York City (NYC), and San Francisco (SF). Each row represents a different city and each column corresponds to a different forecasting model. Stacked bars show the contribution of three CRPS components: overprediction (red), underprediction (green), and dispersion (blue), for each epidemic phase.

A notable divergence between the synthetic and empirical results concerns the EXPI model, which performed competitively in MSM-like simulations but showed substantial overprediction in most cities, particularly in early epidemic phases (Fig 5). In simulated networks, $R_{t}$ declines systematically with cumulative attack rate as high-degree nodes are mechanistically depleted, providing a clear signal from which the attenuation parameter δ can be accurately estimated. In real outbreaks, early epidemic dynamics may be dominated by more stochastic fluctuations in case counts, obscuring the relationship between $A_{t}$ and $R_{t}$ and leading to a poorly identified and underestimated δ. The model consequently fails to anticipate the true pace of epidemic decline, producing forecasts that substantially over-predict incidence. Consistent with this explanation, EXPI performance improved markedly at mid- and later- epidemic phases (4–10), once sufficient data had accumulated to better constrain the attenuation parameter.

The *SIG* model offered superior accuracy across epidemic phases in each city. Visual inspection of city-specific forecasts corroborated these trends, with the sigmoid model performing well overall, but notably overshooting early case trajectories in New York (Fig 6) and undershooting early case counts in Madrid.

**Fig 6:**
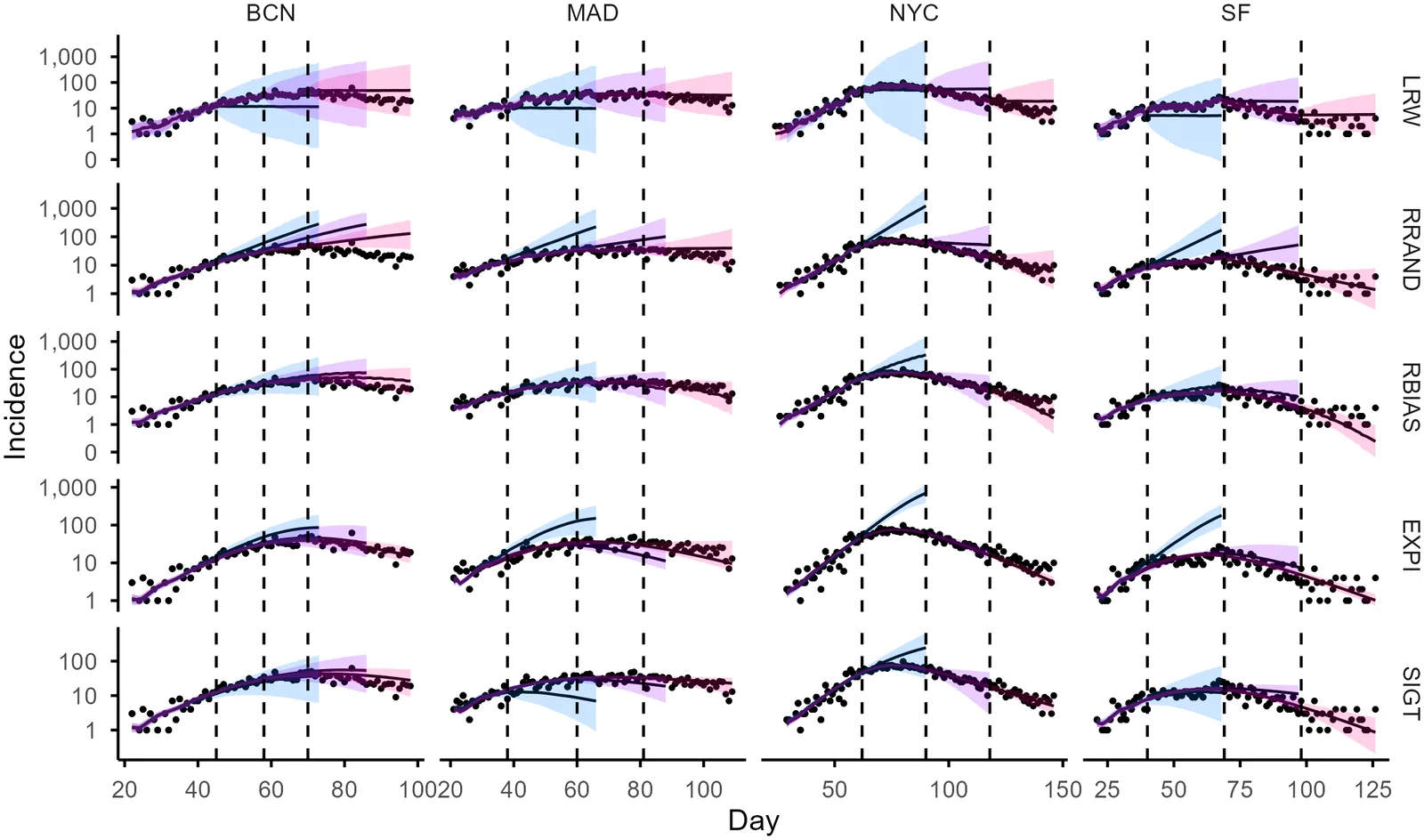
City-level forecasts at three epidemic phases for four epidemic models. Each column corresponds to a city (Barcelona, Madrid, New York City, San Francisco), and each row corresponds to one of four forecasting models (LRW, RRAND, RBIAS, SIGT). Within each panel, model forecasts are shown for three epidemic phases: phase 2 (blue), phase 6 (purple), and phase 10 (pink). Solid black lines represent the median forecast trajectory, while shaded regions denote uncertainty bands. Observed case data are plotted as black points.

## Discussion

In this study, we demonstrate that a modest extension to the classical renewal equation - incorporating a functional attenuation in the effective reproduction number - substantially improves forecast accuracy in settings characterized by heterogeneous and overdispersed contact structures, such as MSM networks. Across both synthetic and real-world outbreaks, the *SIG* model consistently outperformed statistical baselines and standard mechanistic approaches. Its flexible attenuation of $R_{t}$, while remaining computationally simple and interpretable offers a promising direction for improving epidemic forecasting frameworks.

Our results align closely with empirical estimates of the effective reproduction number from recent mpox outbreaks in MSM populations outside endemic regions. For example, estimates from Spain [25] and the United Kingdom [45] show a sharp early decline in $R_{t}$, followed by a gradual tapering–-a trajectory that closely mirrors the sigmoid-shaped attenuation assumed in our model. Estimates from New York showed a small increase in R before a similar sigmoidal-shaped decline [46], which also explains over-prediction of multiple models for the early epidemic phases in New York. The *SIG* model remained the best model when moving from simulated to empirical data, remaining robust despite vaccination programmes which likely had an additional impact in the real world that was not considered in our simulated data [45, 46]. While our simulations suggest this functional form captures network-driven susceptible depletion, such depletion was the only driver of decline, so the simulation comparison should be read as establishing relative forecast skill under depletion-driven dynamics rather than as confirming depletion as the operative mechanism in real outbreaks. In the real-world outbreaks other factors such as behavioural changes and vaccination were also operating and could equally account for the observed model performance. Disentangling these mechanisms would require additional data sources, such as longitudinal information on partner numbers.

One important limitation of the attenuated renewal models is their inherent assumption that the effective reproduction number *R*(*t*) undergoes monotonic attenuation over time. In our implementation, this is enforced by constraining the attenuation rate δ to be positive, effectively ruling out scenarios in which transmission intensity increases during the early epidemic phases. While this assumption matches epidemiological theory that reproduction numbers decline due to depletion of susceptibles, it will fail in contexts where *R*(*t*) increases for other reasons. For example, suppression of contacts early in an ou*t*break due to heightened awareness or public health messaging could be followed by resurgence, causing models with enforced attenuation to underestimate future incidence. This highlights the importance of triangulating forecasts with independent indicators, such as behavioral surveys, to contextualise epidemic trends.

A further limitation concerns the generation time distribution. The simulation framework explicitly distinguishes between one-time and recurrent partnerships, which imply different generation time distributions: contacts through the one-time partner network follow a uniform distribution, while recurrent partnerships produce geometrically distributed generation times reflecting repeated exposure. However, all forecasting models apply a single fixed generation time distribution throughout the epidemic. This is a meaningful simplification, because the relative contribution of each partnership type shifts over the epidemic course: early spread is driven predominantly by the one-time partner network, while recurrent partnerships account for a growing proportion of transmission as high-degree nodes are depleted. The effective generation time distribution therefore changes dynamically during an epidemic, and a static approximation may introduce bias into renewal model estimates of $R_{t}$, particularly in later phases when the partnership mix has shifted substantially. Extending forecasting models to accommodate a time-varying or mixture generation time distribution represents an important direction for future work.

More broadly, the network configuration studied here may apply beyond MSM contexts. For example, the contemporaneous mpox outbreak in the Democratic Republic of Congo [47] appears to involve both social and sexual transmission, also implying the existence of two overlapping networks. Likewise, respiratory infections such as SARS-CoV-2 may spread through layered contact structures, e.g., close, repeated interactions within households and more dispersed contacts in community or workplace settings. Exploring the generalizability of attenuated renewal models to such settings, particularly where contact heterogeneity is high, represents a valuable direction for future research.

The forecasting improvements observed here are likely to generalize beyond MSM epidemics to other infectious diseases that exhibit strong contact heterogeneity. Attenuated renewal models are particularly well-suited for contexts where early epidemic slowdown is driven by the exhaustion of high-risk individuals. In contrast, their benefits may be less pronounced in settings with more homogeneous contact distributions or dominant external drivers (e.g., seasonality). Identifying such structural features early in an outbreak could support the selection of appropriately tailored modelling frameworks.

The framework explored here is based on two overlapping networks: a one-time partner network, which drives early epidemic spread due to its high edge overdispersion, and a main-partner network, which becomes more influential as the epidemic progresses. This is likely why the SIG model performs best: it is able to flexibly and smoothly model the transition in $R_{t}$ from the high intrinsic value in the one-time network through to the low value in the recurrent network (Fig 4).

Our network simulations inevitably rely on simplifying assumptions. We specify particular forms for degree and role assortativity, and assume static networks with fixed recovery durations. In reality, epidemic trajectories are shaped by dynamic behaviors, individual turnover, episodic behaviour, and adaptive responses that are not explicitly captured here. Furthermore, although behavioral change was not explicitly incorporated, some of its effects may be indirectly absorbed through parameter fitting. We also restrict transmission to anal sexual intercourse, which implies that connections between individuals who are exclusively receptive and exclusively insertive are not permitted in the network. In practice, not all sexual contact between MSM involves anal intercourse, and such connections may therefore occur in real sexual networks [48]. We also assumed no delays in reporting which would necessitate back-filling case counts via nowcasting, for which a robust literature already exists (e.g., [11]).

While widely used tools like EpiNow2 [49], Epinowcast [50, 51] and EpiEstim [34] provide flexible, real-time estimates of $R_{t}$, they typically do not account for structural features such as contact heterogeneity or the preferential early depletion of high-degree individuals. As a result, forecasts generated by or based on estimates from these models may systematically overpredict when such depletion effects are present, particularly in networks with high transmission overdispersion. The simplicity and low data demands of attenuated renewal models make them practical for operational use in real-time outbreak response. Because they require only incidence data and an estimate of the generation time distribution, these models can be deployed early in an outbreak, before detailed behavioral or contact-tracing data are available. Their mechanistic structure also allows for interpretability in decision-making, helping public health authorities distinguish between transient noise and meaningful epidemic slowdowns.

## Supporting information

S1 TableFixed inputs and estimated parameters by model. LN(μ,σ) denotes a log-normal prior with log-scale mean μ and standard deviation σ; $N_{T}(\mu ,\sigma )$ denotes a truncated normal; [*a*, *b*] denotes truncation bounds.Priors for estimated parameters are identical across the synthetic and empirical analyses.(XLSX)

S1 FigValidation of realised network properties across 100 MSM-like network simulations.Each panel shows the pairwise relationship between input parameters and directly measured network properties across the combined (main and one-time) partner network. Input parameters are compared against measured network summary statistics in all cases except α, where we demonstrate parameter recovery against the fitted degree sequence exponent. Diagonal panels show the marginal distribution of each variable. Lower triangle panels show scatter plots of each pair. Upper triangle panels show Spearman correlation coefficients, coloured by direction (blue: negative, red: positive) with significance stars (***p < 0.001, **p < 0.01, *p < 0.05). Input and measured values are shown for the degree distribution exponent (α), estimated by maximum likelihood under a truncated power-law, degree assortativity kernel (ζ), measured as the Spearman correlation of degrees across connected node pairs, versatile assortativity parameter (ρ, measured as the proportion of edges incident to at least one versatile node that connect two versatile nodes), and spatial kernel scale (ϵ). Additional measured-only properties are mean proximity ($\bar{d}$, defined as the negative mean Euclidean distance between connected node pairs such that higher values indicate greater spatial clustering) and global clustering coefficient (C, the ratio of closed triangles to connected triples). Strong correlations between input and measured values for all directly controlled parameters (|r| > 0.90) confirm that the generative model reliably produces networks with the intended structural properties.(TIF)

S2 Fig Day-of-week adjustment for New York City (left) and San Francisco (right) case series. Upper panels show raw daily reported case counts; lower panels show series adjusted by dividing by city-specific weekday multipliers estimated via a quasi-Poisson generalised linear model with a log link. In New York City, the raw series exhibits pronounced weekly oscillations arising from lower diagnostic and administrative activity at weekends; these are substantially attenuated following adjustment. In San Francisco, the unadjusted and adjusted series are near-identical, indicating no systematic day-of-week reporting artefact. Estimated weekday multipliers are reported in S2 Table.
(TIF)

S2 Table Estimated weekday reporting multipliers for New York City and San Francisco, derived from quasi-Poisson generalised linear models regressing daily case counts on day of week. Values below 1 indicate suppressed reporting relative to the weekly mean; values above 1 indicate elevated reporting. Observed case counts were divided by the corresponding multiplier to yield the adjusted series.
(XLSX)

S3 Table Mean CRPS scores by model and network at forecast week 0 (days 1–7). (XLSX)

S4 Table Mean CRPS scores by model and network at forecast week 1 (days 8–14). (XLSX)

S5 Table Mean CRPS scores by model and network at forecast week 2 (day 15–21). (XLSX)

S3 Fig Average rank of forecasting models across cities and epidemic phases. Each point represents the mean rank of a model according to its forecasting accuracy measured by Continuous Ranked Probability Score (CRPS), calculated across all epidemic phases and [five] cities. Ranks are shown separately for performance on the linear scale (red) and log scale (blue), with lower values indicating better performance. Horizontal bars denote the interquartile range (IQR) of ranks across all city-phase combinations.
(TIF)
