# Supplementary material for: Forecasting of infectious disease epidemics in sexual networks using attenuated renewal processes
Source: PLoS Comput Biol. 2026 Oct 13;22(10):e1013987. doi: 10.1371/journal.pcbi.1013987 (PMC13686288; doi:10.1371/journal.pcbi.1013987)
Supplement: Supplementary file 1 [file pcbi.1013987.s010.pdf]

Response to Reviewers

PCOMPBIOL-D-26-00282

Forecasting of Infectious Disease Epidemics in Sexual Networks Using Attenuated Renewal Processes

William D. Green, Christopher Overton, Thomas Ward, Ciara McCarthy, Sebastian Funk

Dear Editors,

Thank you for the opportunity to revise our manuscript. We are grateful to the reviewers for their careful reading and constructive comments. The revised manuscript has undergone substantial revision, and we believe it is significantly stronger as a result. The principal changes are as follows.

- We have streamlined the model comparison to five forecasting approaches (LRW, RRAND, RBIAS, EXPI, SIGT), removing two models from the original submission and adding a non-zero floor to EXPI to better reflect the biology of residual transmission through recurrent partnerships.

- We have added a new study design figure (Figure 1) to clarify the paper methodology in response to Reviewer 2's comments, and have substantially expanded the network generation section.

- We have added a supplementary figure validating that variation in input parameters produces the expected variation in directly measured network properties.

- Finally, simulation parameters have been updated based on insights from the literature, with ranges explored explicitly sourced. Relative model performance is conserved across the revised model set.

Below we provide a point-by-point response to each comment, quoting revised text where applicable.

Best wishes,

Will

--

Note: A tracked-changes PDF (diff.pdf) is included alongside this resubmission. This document was generated automatically using latexdiff and highlights additions and deletions between the original and revised manuscripts. Please note two limitations of this document:

- **Tables:** The latexdiff tool does not handle complex table environments reliably. Changes within tables may not render correctly in the diff. We recommend comparing the relevant tables in the original and revised submissions directly; all substantive table changes are described in the point-by-point response below.

- **Citations and cross-references:** The diff compilation process does not resolve citations or internal cross-references, which appear as (?) throughout. These are correct in both the original and revised manuscripts.

Reviewer 1

Major Comments

Major Comment 1: Network generation is underdescribed and unvalidated

Reviewer comment:

> *The network generation model is arguably the most novel methodological contribution of the paper, yet it receives less attention than almost any other component. The authors combine scale-free degree distributions, degree assortativity, role assortativity, spatial mixing, and a recurrent/one-time partner overlay into a single generative framework — but there is almost no validation that the realised networks actually exhibit the intended properties across the range of parameter combinations sampled. The authors must demonstrate that realised networks behave as intended. Distributional summaries of key network properties — degree distribution shape and tail behaviour, assortativity coefficients, clustering — should be measured directly from the generated networks, not inferred from generative parameters. Cloud plots or scatter-plot matrices showing the joint distribution of realised network properties across the 100 MSM-like realisations would be highly informative and should be included. It is also unclear whether this generative model is novel or adapted from prior work. There are very few references in this section, and if the model is new it requires considerably more exposition and justification. Figure 1 should include quantitative network metrics (mean degree, clustering coefficient, assortativity coefficient) alongside the visual representations, which alone are insufficient.*

Response:

We thank the reviewer for this important comment. We have substantially expanded the network generation section and added a new supplementary figure (Figure 8) showing the joint distribution of realised network properties — degree distribution tail exponent, degree assortativity coefficient, role assortativity, mean clustering coefficient, and mean degree — across all 100 MSM-like realisations via a scatter-plot matrix. These directly-measured properties confirm that the generative model successfully spans the intended parameter space. We have also clarified in the Methods that the network generation procedure is a novel combination of components adapted from established approaches. Figure 2 (formally Figure 1) has been updated to include the mean degree, global clustering coefficient, and degree assortativity coefficient for each representative network shown.

Changes made:

Added Supplementary Figure S1: a scatter-plot matrix of input parameter ranges and directly-measured network properties (degree exponent, degree assortativity, role assortativity, clustering coefficient, mean degree) across all 100 MSM-like network realisations. Updated Figure 2 caption and panels to report quantitative network metrics. Expanded the network generation Methods section with references to component methods and explicit acknowledgement of the novel combination.

Major Comment 2: Disconnected network components are not addressed

Reviewer comment:

> *The authors do not address what happens when generated networks fragment into disconnected components, which is a well-known consequence of sparse or highly assortative networks. This is not a minor technical detail. The epidemic seeding strategy, the effective population size, and the interpretation of susceptible depletion all depend critically on whether transmission is constrained to the giant connected component or can propagate between components. The authors should report the distribution of component sizes and the relative size of the largest connected component across realisations, and explicitly state whether epidemics are seeded within the giant component.*

Response:

We thank the reviewer for raising this point. Considering the component structure across all network realisations (with each network containing the partnerships realised over 2 years), the vast majority of nodes belong to the giant connected component (GCC) in all cases: the GCC comprises on average 86.2% of nodes across the 100 MSM-like realisations (range: 74.1–93.3%). Isolated components were small, never exceeding 1% of the overall number of nodes. We have added additional text to describe the relative size of the GCC with the second largest connected component across realisations. All epidemics are seeded within the giant connected component; this is now stated explicitly in the Methods.

Changes made:

Added text to the epidemic simulation Methods section:

"Generated networks were not fully connected; across the 100 MSM-like realisations, the largest connected component contained a median of 86.2% of nodes (IQR: 83.3-88.2%, range: 74.1-93.3\%). The second largest component never exceeded 1\% of the size of the giant component, indicating that remaining nodes were largely isolated or in very small clusters rather than large competing sub-components. Two features of the epidemic simulation design (detailed below) ensured that epidemics propagated through the giant component in practice. First, the epidemic was seeded probabilistically in nodes with at least 10 connections, which are overwhelmingly located within the giant component given the heavy-tailed degree distribution. Second, simulations were rerun until a minimum of 500 cases were observed, effectively discarding any realisations in which the epidemic failed to propagate beyond a small isolated subcomponent."

Major Comment 3: Unequal numbers of realisations across network classes are not justified

Reviewer comment:

> *The study uses 30 homogeneous, 30 heterogeneous, and 100 MSM-like network realisations. No justification is provided for this asymmetry. Since primary comparisons are made across network types, unequal sample sizes introduce inconsistency in statistical power. The authors should either justify this design explicitly — for example via a power or precision argument — or adopt equal sample sizes throughout (e.g. 100 per class).*

Response:

We thank the reviewer for this comment. The asymmetry reflects the complexity of the parameter space rather than statistical design: the homogeneous and heterogeneous networks each have far fewer free parameters (5–6) and consequently require fewer realisations to characterise their behaviour. The 100 MSM-like realisations were chosen to provide adequate coverage of a substantially higher-dimensional parameter space (12 parameters sampled via LHS). We have added this justification to the Methods. We note that the primary scientific comparisons in the paper are across models (not across network types), and all model comparisons within a network class use the same number of realisations; the across-class asymmetry therefore does not introduce the kind of power inconsistency the reviewer is concerned about for those comparisons. Nonetheless, we accept that this should be stated explicitly rather than left implicit.

Changes made:

Added the following sentence to the synthetic analysis Methods: "The number of realisations differs across network classes to reflect their respective parameter space dimensionality). Homogeneous and heterogeneous networks have fewer free parameters and serve primarily as benchmarks under simplified structural assumptions so 30 realisations are used to characterise model performance in these settings. MSM-like networks are parameterised by many more structural properties, motivating a larger number of realisations (n = 100) to adequately cover the higher-dimensional parameter space and ensure robust comparisons across network configurations."

Major Comment 4: The SIGT model, the paper's central contribution, is poorly motivated

Reviewer comment:

> *Equation 14 is presented without any mechanistic or intuitive explanation. Why a reverse-sigmoid? What biological or network process does this functional form represent? What does the floor parameter R_floor correspond to in terms of network dynamics — is it the reproduction number in the recurrent partner network once one-time contacts are exhausted? The authors gesture at this interpretation in the Discussion but it must appear in the Methods where the model is introduced. The authors should also explore or at minimum discuss alternative functional forms for the attenuation. A reverse-sigmoid is one of many plausible choices; others include a generalised logistic, a Gompertz decay, or a piecewise linear approximation. The choice should be explicitly defended.*

Response:

We thank the reviewer for this substantive comment. The interpretation of the sigmoid function as captured a smooth transition from of the two-network transition now appears in the Methods. Regarding alternative functional forms: we now mention a Gompertz decay as a possible alternate formulation that could be considered; a piecewise linear approximation with discontinuity in first derivative is less parsimonious. We have also adapted the EXPI model parameterised by attack rate rather to include a floor, making it more comparable with the sigmoid method.

Changes made:

The methods now contain the following text.

A reverse-sigmoidal decay in R as a function of time ($SIGT$). The reverse-sigmoid functional form is motivated by the two-network structure of MSM sexual contact networks. Early in an epidemic, transmission is driven primarily by the one-time partner network, where high edge overdispersion results in rapid infection of high-degree nodes and a correspondingly high effective reproduction number. As these highly-connected individuals are depleted from the susceptible population, transmission increasingly reflects the recurrent partner network, where connectivity is lower and more homogeneous. The sigmoid was chosen as it captures a smooth transition from the high effective reproduction number characteristic of the early epidemic — driven by the one-time partner network — to the lower reproduction number of the later epidemic, dominated by recurrent partnerships, while yielding directly interpretable parameters. Other monotonically decreasing smooth functional forms, such as the Gompertz decay or generalised logistic, could in principle be considered but are left for future work.

Major Comment 5: Fitted versus fixed parameters are never distinguished

Reviewer comment:

> *This is a significant transparency problem that runs throughout the paper. The paper never explicitly distinguishes between parameters that are fitted (estimated from data via HMC), parameters that are fixed from external sources, and parameters that are set by the simulation and therefore known when forecasting. Specifically: (i) the generation time distribution ω(τ) — is this fixed from the literature or estimated? (ii) population size N — is it fixed, estimated, or marginalised over in the empirical analysis? (iii) the incubation period in the empirical component — fixed or fitted? (iv) the initial number of infecteds I(0) — the prior is listed, but it is unclear whether this quantity is observable or latent. The authors must include a clear table distinguishing fixed from estimated parameters, presented separately for the synthetic and empirical analyses, with explicit justification for all fixed values.*

Response:

We thank the reviewer for identifying this important transparency gap. We have added Supplementary Table 3 which explicitly separates parameters into estimated or fitted, and does so for both the empirical analysis and the simulation. To address the specific queries: (i) the generation time distribution for mpox $\omega(\tau)$ is fixed from the literature, and now sourced in the references; (ii) population size N is fixed at the estimated MSM population size for each city (now referenced), or as the network size in the simulations; (iii) the fitted terms include terms pertaining to the reproduction number, the initial infected, the overdispersion in the count data and the attenuation rate. I(0), the seed infections, is a latent parameter estimated via HMC with the prior stated in Table 3.

Changes made:

Added Supplementary Table 1 distinguishing fitted, fixed, and simulation-determined parameters for both synthetic and empirical analyses, with sources for all fixed values. Added clarifying text to the Fitting Approach section.

Major Comment 6: The seven-model comparison is poorly justified

Reviewer comment:

> *Several comparator models (EXPI and POWI in particular) perform so poorly — often by an order of magnitude relative to SIG — that their inclusion adds little analytical value and dilutes the key comparisons. The paper would benefit from a more principled selection: a pure statistical baseline (LRW), the classical renewal model (RRAND), and the most credible alternative attenuation approach (RBIAS). If all seven are retained, the authors must motivate why each is epidemiologically plausible in this setting. Currently the Forecasting Approaches section offers almost no justification for any model beyond a single reference.*

Response:

We thank the reviewer for this comment and agree that the original model set was too large and included comparators that were too weak to provide meaningful discrimination. We have reduced the model set from seven to five by dropping POWI, EXPT, as these were consistently the poorest performers and the least epidemiologically motivated. The EXPI model was updated to include a floor in R, making it a more fair comparison to the SIGT model. The retained five models can each be justified by a distinct perspective on how the reproduction number decays over the course of an epidemic: LRW serves as a purely statistical baseline with no mechanistic structure; RRAND is the canonical renewal model in which R_t evolves as a driftless random walk; RBIAS extends RRAND with a principled downward drift, providing the most credible purely statistical attenuation; SIGT parameterises attenuation through calendar time, capturing the hypothesis that epidemic decline is driven by a temporal transition from highly connected nodes, to low connected nodes; and EXPI - reformulated with a floor parameter - parameterises attenuation through cumulative attack rate, capturing the hypothesis that decline is driven by connectivity-biased population-level depletion. Together these five models span the space from agnostic (LRW, RRAND) through principled statistical attenuation (RBIAS) to mechanistically motivated attenuation indexed by time (SIGT) and by incidence (EXPI). We have rewritten the Forecasting Approaches section to make this structure explicit.

Changes made:

Reduced model set from seven to five by dropping POWI, EXPT, and the original floor-free EXPI. Reformulated EXPI to include a floor parameter analogous to that in SIGT. Rewrote the Forecasting Approaches section to clearly motivate each retained model.

Major Comment 7: Day-of-week adjustment for New York data is insufficiently justified

Reviewer comment:

> *The quasi-Poisson GLM approach to correcting weekend reporting artefacts in the NYC data is stated but not adequately motivated. It is not clear why this specific approach is appropriate, how large the estimated weekday effects are, or whether the adjustment meaningfully changes the shape of the epidemic curve. The authors should show before-and-after plots of the raw and adjusted NYC time series, and report the estimated weekday multipliers. The same level of scrutiny should be applied to the San Francisco data.*

Response:

We thank the reviewer for this comment and agree. We had previously applied the day-of-week adjustment only to NYC, where weekday effects were significant, but have now applied the same adjustment procedure to San Francisco as well. We fit a generalised linear model with quasi-Poisson errors and a log link to each series, regressing daily case counts on day of week. The quasi-Poisson family is appropriate here because it accommodates the overdispersion typical of routine surveillance data, and the log link imposes a multiplicative structure whereby weekend suppression acts as a constant proportional scaling of true incidence regardless of epidemic magnitude - a more defensible assumption than an additive correction. We have added a supplementary figure (Supplementary Figure 2) showing the raw and adjusted epidemic curves for both cities, and a supplementary table reporting the estimated day-of-week multipliers (Supplementary Table 2). For NYC the effects were substantial; for San Francisco the adjustment had more limited impact on the resulting curve.

Changes made:

Applied day-of-week correction to San Francisco data in addition to NYC. Added supplementary figure showing raw vs. adjusted epidemic curves for both cities (Supplementary Figure 2). Added supplementary table of day-of-week multipliers (Supplementary Table 2). Expanded the Data section with the following text, also justifying the quasi-Poisson approach:

"Data for San Fransisco is taken from the publicly available surveillance dashboard by the San Francisco Department of Public Health (SFDPH) \cite{SF_mpox}. The dataset reports incident cases by date of report. Data for New York was sourced from the New York City Department of Health and Mental Hygiene (NYC DOHMC) \cite{nychealth_monkeypox_data}, with cases reported by date of diagnosis. Both datasets are therefore potentially liable to day-of-week reporting artefacts arising from clinic operating hours and administrative processing. We applied a diagnostic check to each series by fitting a generalised linear model with quasi-Poisson errors and a log link (appropriate for overdispersed count data) regressing daily case counts on day of week. Quasi-Poisson errors accommodate the overdispersion typical of routine surveillance data, and the log link imposes a multiplicative structure whereby weekend suppression acts as a constant proportional scaling of true incidence regardless of epidemic magnitude. We apply this correction as a pre-processing step rather than adjusting within the models in order to keep the observation model identical across the five approaches and support comparison of the approach to forecasting latent dynamics. For New York City, day-of-week effects were substantial (Supplementary Figure \ref{fig:dow_adjustment}, Supplementary Table \ref{tab:dow_multipliers}). For San Francisco, a similar day-of-week correction was applied but with more limited impact on the resulting epi-curve (Supplementary Figure 2, Supplementary Table 2). We divided observed daily counts by the corresponding weekday-specific factor to yield an adjusted series preserving the underlying epidemic trend."

Major Comment 8: Forecast evaluation choices are unmotivated

Reviewer comment:

> *The choice to forecast 28 days ahead and evaluate only on days 22–28 is never explained. Why 28 days? Why evaluate only the final week of the horizon rather than performance across the full forecast window? Similarly, the choice of ten equally spaced*

forecast time points is stated without explanation of what the temporal gap between them is in absolute terms, or how this relates to the duration of individual epidemic curves.

Response:

We thank the reviewer for raising this point; we agree that these choices required explicit justification, which was absent from the original submission. Forecasts were generated at ten equally spaced time points spanning the period from when 5% to 95% of all infections had occurred in each simulation, ensuring coverage of the full epidemic trajectory from early growth through decline. The 28-day horizon was chosen to reflect the timescale over which public health authorities must plan resource allocation and intervention responses, and is consistent with established infectious disease forecasting frameworks (Cramer et al. 2022; Sherratt et al. 2023). Performance was evaluated on days 22–28 (the final week of the horizon) rather than the full window because near-term predictions (days 1–7) tend to be accurate across all models as they extrapolate from data close to the forecast cutoff, making early-horizon performance uninformative for model discrimination; longer-horizon accuracy is the more operationally relevant quantity. For completeness, we have added a Supplementary Table showing CRPS decomposed by individual forecast week (days 1–7, 8–14, 15–21).

Changes made:

Added explicit justification for the 28-day horizon, the days 22–28 evaluation window, and the ten equally spaced forecast time points to the Forecast Evaluation section. Added supplementary table showing CRPS decomposed by forecast week. The revised text reads:

"For each simulation, forecasts were generated at ten equally spaced time points spanning the period from when 5\% to 95\% of all infections had occurred, corresponding to a median inter-forecast interval of 8.5 days (95\% interval across simulations: 4.1-16.8 days). This spacing ensured that forecast origins were distributed across the full epidemic trajectory - encompassing growth, peak, and decline - rather than concentrated in any single phase. Each model predicted incidence 28 days into the future from each forecast cutoff, a horizon chosen to reflect the timescale over which public health authorities must plan resource allocation and intervention responses, and consistent with established infectious disease forecasting frameworks \cite{Cramer2022, Sherratt2023}. Model performance was assessed based on predicted incidence during days 22-28 post-cutoff (the final week of the horizon) rather than the full window. Near-term predictions (days 1-7) tend to be accurate across all models as they extrapolate from data close to the forecast cutoff, making early-horizon performance uninformative for model discrimination; longer-horizon accuracy is the more operationally relevant quantity, where decisions must be made well in advance of projected peaks or troughs. For completeness, mean CRPS decomposed by individual forecast week (days 1-7, 8-14, 15-21, and 22-28) is provided in the supplementary material (Supplementary Tables 3-5)."

Minor Comments

Minor Comment 1

Reviewer comment:

Line 161: "This includes one statistical models" — grammar error, should be "one statistical model."

Response:

Corrected.

Changes made:

"one statistical models" corrected to "one statistical model" on line 161.

Minor Comment 2

Reviewer comment:

> *Equation 3 is presented as a free-floating display rather than being integrated into the preceding paragraph. It should be introduced inline and should end with a full stop or comma as appropriate to the surrounding sentence.*

Response:

Corrected. Equation 3 is now introduced by the preceding sentence and ends with a full stop.

Changes made:

Equation 3 is now introduced with "...the spatial kernel is given by:" and ends with a full stop.

Minor Comment 3

Reviewer comment:

> *The caption of Table 2 should briefly define each of the seven model abbreviations rather than expecting readers to cross-reference back to the Methods.*

Response:

We have added definitions of all model abbreviations to the Table 2 caption.

Changes made:

Table 2 caption now includes: " Models considered are log random walk (LRW); a renewal model with random walk in R_t (RRAND); a renewal model with a biased random walk in R_t (RBIAS); a renewal model with exponential attenuation of R_t to a R_{floor} (EXPI); and a renewal model with sigmoidal attenuation of R_t (SIGT)"

Minor Comment 4

Reviewer comment:

> *Figure 2 requires considerably more explanation in its caption. Readers unfamiliar with CRPS decomposition will not understand what a bar dominated simultaneously by large red (overprediction) and green (underprediction) components indicates. The y-axis should also be labelled with units.*

Response:

We have substantially expanded the Figure 2 caption to explain the CRPS decomposition and adjusted the y-axis units.

Changes made:

Expanded Figure 2 caption with an explanation of CRPS decomposition interpretation. Figure caption now reads:

"Decomposition of the Continuous Ranked Probability Score (CRPS) for the MSM-like networks into overprediction (red; forecast distribution places excess mass above the observation), dispersion (green; penalty for forecast spread, reflecting uncertainty in the predictive distribution), and underprediction (blue; forecast distribution places excess mass below the observation) for MSM-like network simulations ($n = 100$). Results are shown separately for the linear scale (top; y-axis in units of cases) and log scale (bottom; y-axis in units of log cases). The x-axis denotes epidemic phase, where earlier phases correspond to forecasts made during epidemic growth and later phases to forecasts made near or after the epidemic peak. A bar dominated simultaneously by large overprediction and underprediction components indicates a model that is highly variable across simulations - alternately projecting too high and too low - without sufficiently wide forecast intervals to capture this variation. For example, RRAND shows consistently large overprediction on the linear scale, particularly at early epidemic phases, reflecting the tendency of an unconstrained random walk to project continued exponential growth when the true epidemic is declining. By contrast, SIGT produces small bars across most phases, with residual error attributable primarily to dispersion, indicating well-centred forecasts with appropriately calibrated uncertainty rather than systematic directional bias."

Minor Comment 5

Reviewer comment:

> *In Figure 5, the caption states that phases are shown in red, green, and blue, but these colours do not correspond to what is displayed in the figure. This must be corrected.*

Response:

Corrected. The caption has been updated to accurately describe the colours used in the figure (now Figure 6).

Changes made:

Figure 5/6 caption corrected to accurately describe the colour scheme used in the displayed figure.

Minor Comment 6

Reviewer comment:

> *The figures appear to be placed at considerable distance from their corresponding discussion in the text. The Results section largely restates Table 2 numerically without sufficient interpretation.*

Response:

We have revised the Results section to more closely integrate the figures into the narrative, with explicit pointers to each figure at the point of relevant discussion. The numerical restatement of Table 2 has been reduced and replaced with a more interpretive narrative highlighting the key findings.

Changes made:

Results section revised to integrate figures more closely with the narrative. Reduced numerical restatement of Table 2 in favour of interpretive text.

Minor Comment 7

Reviewer comment:

> *The prior distributions in Table 3 (supplementary) are not discussed or defended in the main text. For a Bayesian model comparison, prior sensitivity is a legitimate concern and should at minimum be acknowledged.*

Response:

We thank the reviewer for this comment. We have added a paragraph to the Fitting Approach section motivating the key prior choices. Priors were chosen to be weakly informative, centred on epidemiologically plausible values while allowing the likelihood to dominate inference where data are sufficient. The prior on R_0 has a median of 10 with a 95% interval of 4.6–21.9, permitting a broad range of early exponential growth rates; the overdispersion parameter φ is centred at 30 (95% interval 11.3–79.9), reflecting moderate overdispersion consistent with routine surveillance data; and priors on attenuation parameters were chosen to permit a broad range of epidemic trajectories from rapid to gradual decline. Importantly, shared parameters (R_0, $I(0)$, φ) use identical priors across all renewal models, ensuring that performance differences in model comparison reflect model structure rather than prior specification.

Changes made:

Added the following paragraph to the Fitting Approach section: "Prior distributions were chosen to be weakly informative, centred on epidemiologically plausible values while allowing the likelihood to dominate inference where data are sufficient (Table 3). The prior on R_0 was log-normal with median 10 and a 95% interval spanning 4.6–21.9, allowing for a broad range of early exponential growth. The overdispersion parameter φ is centred at 30, but again this was weakly informative with 95% bounds of 11.3 and 79.9, suggesting moderate overdispersion. Priors on attenuation parameters were chosen to permit a broad range of epidemic trajectories, from rapid to gradual decline. Shared parameters (R_0, $I(0)$, φ) use identical priors across all renewal models, ensuring that performance differences reflect model structure rather than prior specification."

Minor Comment 8

Reviewer comment:

> *Lines 205–206 state that CRPS reduces to mean absolute error for deterministic forecasts as a way of introducing the metric intuitively. However, all models in this paper produce probabilistic forecasts. This framing is therefore misleading and should be replaced with a more relevant intuitive description.*

Response:

We thank the reviewer for this comment and agree that the reference to deterministic forecasts was misleading in the context of a study using exclusively probabilistic models. We have revised the introduction to CRPS to describe the metric directly in terms of probabilistic forecast evaluation. The revised text describes how CRPS rewards distributions that place high probability mass close to the observed value, jointly assessing calibration and sharpness, and explains the decomposition into overprediction, underprediction, and dispersion components. The reference to mean absolute error has been removed.

Changes made:

Replaced the deterministic-forecast framing with the following text:

"The CRPS rewards forecast distributions that place high probability mass close to the observed value: a sharp distribution well-centred on the observation scores well, while a distribution that is poorly centred, unnecessarily wide, or both is penalised. The CRPS therefore jointly assesses calibration (whether observations fall in plausible regions of the forecast distribution) and sharpness (whether the distribution is appropriately concentrated). The CRPS can be decomposed into overprediction, underprediction, and dispersion terms, providing insight into whether model errors arise from systematic bias (forecasts being consistently too high or too low) or from insufficiently calibrated forecast uncertainty (too narrow or too wide predictive intervals). We computed CRPS on both the linear and logarithmic scales as recommended in Bosse et al. (2023); the linear scale facilitates evaluation of absolute features such as peak incidence, while the logarithmic scale provides a measure of relative error allowing more sensitive assessment across different epidemic magnitudes."

Minor Comment 9

Reviewer comment:

> *The paper acknowledges that one-time and recurrent contacts have different generation time distributions (uniform vs. geometric) but uses a single fixed generation time in all forecasting models. This is flagged as a future direction but is a current limitation that should be stated explicitly and prominently in the Limitations section.*

Response:

We agree. We now discuss this more explicitly in the Discussion.

Changes made:

Added text: " A further limitation concerns the generation time distribution. The simulation framework explicitly distinguishes between one-time and recurrent partnerships, which imply different generation time distributions: contacts through the one-time partner network follow a uniform distribution, while recurrent partnerships produce geometrically distributed generation times reflecting repeated exposure. However, all forecasting models apply a single fixed generation time distribution throughout the epidemic. This is a meaningful simplification, because the relative contribution of each partnership type shifts over the epidemic course: early spread is driven predominantly by the one-time partner network, while recurrent partnerships account for a growing proportion of transmission as high-degree nodes are depleted. The

effective generation time distribution therefore changes dynamically during an epidemic, and a static approximation may introduce bias into renewal model estimates of R_t, particularly in later phases when the partnership mix has shifted substantially. Extending forecasting models to accommodate a time-varying or mixture generation time distribution represents an important direction for future work."

Minor Comment 10

Reviewer comment:

> *It is generally not recommended to evaluate forecasts by fitting to cumulative incidence distributions. The authors should clarify whether this is what is intended by their CRPS evaluation setup and, if so, justify why.*

Response:

We thank the reviewer for raising this point, but wish to clarify that our evaluation does not involve fitting to or scoring against cumulative incidence distributions. Rather, for each forecast cutoff, we sum predicted and observed daily incidence over a fixed future window (days 22–28 post-cutoff) and evaluate the CRPS against this weekly total. This is equivalent to scoring a probabilistic forecast of weekly incidence — a standard approach in infectious disease forecasting [Cramer et al. 2022; Sherratt et al. 2023] — and does not share the statistical problems associated with scoring cumulative incidence (e.g., autocorrelation and monotonicity).

Minor Comment 11

Reviewer comment:

> *The Forecasting Approaches section is written exclusively for specialists. It would benefit from at least one or two sentences of background and intuition before the equations, and from additional references motivating each modelling choice beyond the single citation currently provided.*

Response:

We agree. We have added an introductory paragraph to the Forecasting Approaches section providing background and intuition accessible to a broader readership

Changes made:

Added text: "Renewal process models provide a natural framework for short-term epidemic forecasting by expressing current incidence $I(t)$ as a weighted sum of past incidence, with weights given by the generation time distribution ($\omega(\tau)$ \cite{cori2013new}:

\begin{equation}\label{renewal_eq}

I(t) = R_t \sum_{\tau} I(t-\tau) \, \omega(\tau),

\end{equation}

The effective reproduction number R_t summarises current transmission intensity: values above one indicate epidemic growth, values below one indicate decline. Forecasting then reduces to specifying how R_t is expected to evolve beyond the observation window: different assumptions about this evolution define the model comparison in this study."

Reviewer 2

Comment 0

Reviewer comment:

> *At first, I got the impression that the authors consider a stochastic agent-based model. Then, however, there is also a statement on a log-random walk and a renewal model.*

Response:

We thank the reviewer for raising this point and apologise for the lack of clarity in the original manuscript. We have added Figure 1, a schematic overview of the study design, which makes the two-stage structure of the paper explicit. The figure shows that the analysis comprises two parallel pipelines - synthetic network simulations and real-world incidence data - both feeding into the same forecasting and evaluation framework. The left panel illustrates that the stochastic SEIR model operates at the individual node level on the generated contact networks, producing synthetic incidence time series; the right panel shows that the five forecasting models are then fitted to these aggregated time series (and to real-world surveillance data). The two components are therefore distinct: the agent-based simulator is the data-generating mechanism for the synthetic analysis, and the forecasting models are the objects of evaluation. We hope this figure resolves the confusion.

Changes made:

Added Figure 1, a schematic overview of the full study design, illustrating the two-stage structure: (i) network generation and stochastic SEIR simulation as the data-generating mechanism, and (ii) population-level statistical and semi-mechanistic forecasting models fitted to the resulting incidence time series for evaluation.

Comment 1

Reviewer comment:

> *Overall, the section "Forecasting approaches" and Eqs. (7)–(15) do not provide sufficient information to understand the model setup.*

Response:

We thank the reviewer for this comment and agree that the original section was insufficiently detailed. We have substantially revised the Forecasting approaches section. We now open with an introductory paragraph explaining that renewal process models express current incidence as a

weighted sum of past incidence via the generation time distribution (Eq. 7), and that forecasting reduces to specifying how R_t is expected to evolve beyond the observation window — making the model comparison framework immediately transparent to non-specialists.

Changes made:

Added explanatory Figure 1 as a schematic for the work. Rewrote the Forecasting approaches section to include an introductory paragraph on renewal processes accessible to non-specialists; explicit epidemiological motivation for each of the five retained models; mechanistic motivation for the SIGT reverse-sigmoid form; motivation for the EXPI reformulation with a floor parameter; and clarification that all renewal models share Eqs. 9 and 14 as a common backbone.

Comment 2

Reviewer comment:

> *It is also unclear how the probability given in Eq. (1) is normalized.*

Response:

We thank the reviewer for raising this point. We have updated Equation 1 to explicitly show the normalisation constant, making clear that the edge formation probability is normalised over the set of all eligible partners for node i.

Changes made:

Equation 1 now reads: $P(i \leftrightarrow j) = d_j A_{ij} R_{ij} S_{ij} / \Sigma_{l \in \varepsilon_i} d_l A_{il} R_{il} S_{il}$*, where* ε_i *denotes the set of eligible partners for node i (nodes with remaining degree capacity, compatible role, no prior connection, and index l > i).*

Comment 3

Reviewer comment:

> *Furthermore, why is SEIR a suitable model?*

Response:

We thank the reviewer for this comment. The SEIR framework was chosen for its generalisability to a broad range of epidemics rather than being calibrated to any single pathogen. The inclusion of an exposed (latent) compartment is relevant to many common epidemics which can be transmitted by close contact.

Changes made:

The epidemic simulation section now opens with: "Epidemics were simulated using a stochastic SEIR (Susceptible–Exposed–Infectious–Removed) framework on the generated networks, chosen for its generalisability to a broad range of sexually transmitted infections."

Comment 4

Reviewer comment:

The homogeneous network is certainly not realistic. Besides, it is unclear why a uniform degree distribution leads to a homogeneous network. Do the authors mean a regular network where every node has the same degree?

Response:

We thank the reviewer for this clarification. The reviewer is correct: the network we referred to as "homogeneous" is technically a regular network in which every node has an identical degree, not one with a uniform degree distribution. We have corrected the terminology throughout and clarified that the homogeneous network was included purely as a theoretical comparator to isolate the effect of degree heterogeneity, not as a realistic representation of sexual contact patterns.

Changes made:

The relevant sentence now reads: "In the synthetic analysis, we simulate epidemics across three classes of networks: (i) 30 simple regular networks in which every node has an identical degree (henceforth referred to as 'homogeneous' networks), (ii) 30 simple heterogeneous networks with degree distributions drawn from a truncated power-law, and (iii) 100 realistic MSM-like networks characterised by an overlapping one-time and recurrent partner structure."

We have also added: "The homogeneous network was included as a theoretical comparator to isolate the effect of degree heterogeneity, not as a realistic representation of sexual contact patterns."

Comment 5

Reviewer comment:

Table 1 is not informative as it omits information on every network type.

Response:

The blank cells in Table 1 are intentional: parameters not applicable to a given network type are left blank, as the corresponding structural features are absent from those network classes. We have made this explicit in the table caption to avoid ambiguity.

Changes made:

The table is now organised in such a way that shared features are at the top, and the caption now includes: "Parameters not applicable to a given network type are left blank."

Comment 6

Reviewer comment:

Table 1 provides ranges for many parameters. Which were used in the end? Did the authors consider a systematic parameter scan?

Response:

We thank the reviewer for this comment. As stated in the section *Simulation Studies on Synthetic Networks* and in the caption of Table 1 caption, parameter combinations were drawn using Latin

hypercube sampling (LHS) across the ranges specified, providing systematic space-filling coverage of the parameter space without requiring an exhaustive grid search. As this information was present in the caption we have made no additional changes in response to this comment.

Changes made:

No changes made to the manuscript in response to this comment.

Comment 7

Reviewer comment:

> *The truncated power-law is also confusing. How is a range of power-law exponents between -2 and -1.6 justified for a contact network?*

Response:

We thank the reviewer for the comment and agree that additional justification was warranted. The range was motivated empirically based on estimates arising from the Natsal data in Whittles et al.

Changes made:

Table 1 now includes a footnote giving the source for the considered ranges for the degree distribution parameter and the degree upper bound:

"^{a}range estiamted from \cite{whittles2019dynamic}"

Comment 8

Reviewer comment:

> *The idea of spatial proximity does not agree with a network modeling approach. The intention for a spatial embedding is unclear.*

Response:

We thank the reviewer for raising this point and acknowledge that the original text did not make the role of spatial proximity sufficiently clear. Spatial proximity is incorporated as a node-level attribute that influences edge formation probability during network construction — it does not act directly on transmission dynamics. This is a standard approach in spatially-embedded network models, reflecting the empirical finding that sexual partnerships are more likely to form between geographically proximate individuals. We have clarified this in the revised manuscript.

Changes made:

The spatial mixing paragraph now reads: "Spatial proximity \cite{algarin2018spatial} was incorporated as a node-level attribute: each node was assigned coordinates drawn uniformly from the unit square, and the probability of edge formation between nodes i and j was modulated by an exponentially decaying spatial kernel parameterised by ε (equation \ref{spatial_eq}). This reflects the empirical finding that sexual partnerships are more likely to form between geographically proximate individuals, thus the spatial parameter influences network topology during edge formation."

Comment 9

Reviewer comment:

Shouldn't the underlying network have a temporal component?

Response:

We thank the reviewer for the comment and agree on the need for clarification. The network does have a temporal component, which we have now made more explicit in the revised manuscript. One-time partnership edges are each assigned a single contact day drawn uniformly at random over the simulation period, while recurrent partnership edges operate with a daily contact probability p_{rec}. The time-varying contact indicator $c_{ij}(t)$ captures this distinction explicitly in the transmission equation. We have clarified this in both the network generation and epidemic simulation sections.

Changes made:

The network generation section now states: "The one-time network was constructed first, with each edge assigned a single contact day drawn uniformly at random over the simulation period. The recurrent network was constructed second, with an edge between two nodes disallowed in the recurrent network if they were already connected in the one-time network." The definition of $c_{ij}(t)$ now reads: "The contact indicator $c_{ij}(t) \in \{0, 1\}$ equals 1 if nodes i and j had sexual contact at time t and 0 otherwise. For recurrent partnerships, contact at each time step occurs with probability p_{Rec}; for one-time partnerships, contact occurs only on the single day assigned to that edge during network construction."
