# Supplementary material for: Forecasting of infectious disease epidemics in sexual networks using attenuated renewal processes
Source: PLoS Comput Biol. 2026 Oct 13;22(10):e1013987. doi: 10.1371/journal.pcbi.1013987 (PMC13686288; doi:10.1371/journal.pcbi.1013987)
Supplement: Peer review history

# Peer review: Decision Letter 0

**DOI:** 10.1371/journal.pcbi.1013987.r001

**Authors:** Jennifer Flegg, Konstantin B. Blyuss

19 Mar 2026

PCOMPBIOL-D-26-00282

Forecasting of Infectious Disease Epidemics in Sexual Networks Using Attenuated Renewal Processes

PLOS Computational Biology

Dear Dr. Green,

Thank you for submitting your manuscript to PLOS Computational Biology. After careful consideration, we feel that it has merit but does not fully meet PLOS Computational Biology's publication criteria in its currently form. As things stand, both reviewers have raised numerous and serious concerns regarding how things are presented in the manuscript, both in terms of methodology, and with respect to what was actually done. Therefore, we invite you to submit a significantly revised version of the manuscript that properly and comprehensively addresses all the points raised during the review process.

Please submit your revised manuscript by May 19 2026 11:59PM. If you will need more time than this to complete your revisions, please reply to this message or contact the journal office at ploscompbiol@plos.org. When you're ready to submit your revision, log on to https://www.editorialmanager.com/pcompbiol/ and select the 'Submissions Needing Revision' folder to locate your manuscript file.

Please include the following items when submitting your revised manuscript:

* A letter that responds to each point raised by the editor and reviewer(s). You should upload this letter as a separate file labeled 'Response to Reviewers'. This file does not need to include responses to formatting updates and technical items listed in the 'Journal Requirements' section below.

* A marked-up copy of your manuscript that highlights changes made to the original version. You should upload this as a separate file labeled 'Revised Manuscript with Track Changes'.

* An unmarked version of your revised paper without tracked changes. You should upload this as a separate file labeled 'Manuscript'.

If you would like to make changes to your financial disclosure, competing interests statement, or data availability statement, please make these updates within the submission form at the time of resubmission. Guidelines for resubmitting your figure files are available below the reviewer comments at the end of this letter

We look forward to receiving your revised manuscript.

Kind regards,

Konstantin B. Blyuss

Academic Editor

PLOS Computational Biology

Jennifer Flegg

Section Editor

PLOS Computational Biology

**Journal Requirements:**

1) Please ensure that the CRediT author contributions listed for every co-author are completed accurately and in full.

At this stage, the following Authors/Authors require contributions: Chris Overton, Thomas Ward, Ciara McCarthy, and Sebastian Funk. Please ensure that the full contributions of each author are acknowledged in the "Add/Edit/Remove Authors" section of our submission form.

The list of CRediT author contributions may be found here: https://journals.plos.org/ploscompbiol/s/authorship#loc-author-contributions

2) We ask that a manuscript source file is provided at Revision. Please upload your manuscript file as a .doc, .docx, .rtf or .tex. If you are providing a .tex file, please upload it under the item type u2018LaTeX Source Fileu2019 and leave your .pdf version as the item type u2018Manuscriptu2019.

3) Please provide an Author Summary. This should appear in your manuscript between the Abstract (if applicable) and the Introduction, and should be 150-200 words long. The aim should be to make your findings accessible to a wide audience that includes both scientists and non-scientists. Sample summaries can be found on our website under Submission Guidelines:

https://journals.plos.org/ploscompbiol/s/submission-guidelines#loc-parts-of-a-submission

4) Please upload all main figures as separate Figure files in .tif or .eps format. For more information about how to convert and format your figure files please see our guidelines:

https://journals.plos.org/ploscompbiol/s/figures

**Reviewers' comments:**

Reviewer's Responses to Questions

**Comments to the Authors:**

**Please note here if the review is uploaded as an attachment.**

Reviewer #1: Referee Report

Green et al.

“Forecasting of Infectious Disease Epidemics in Sexual Networks Using Attenuated Renewal Processes”

PLOS Computational Biology — Manuscript PCOMPBIOL-D-26-00282

General Comments

This paper addresses a genuinely important and well-motivated problem. The core finding — that a reverse-sigmoid attenuated renewal model consistently outperforms alternatives in overdispersed MSM contact networks — is interesting and potentially useful for real-time outbreak response. The combination of synthetic network simulations and empirical validation across five cities is conceptually the right design, and the use of CRPS on both linear and log scales reflects methodological maturity.

However, the manuscript reads as if written in haste. Methods are presented without motivation or intuition, critical modelling decisions go unjustified, and several components that are central to the paper’s contribution — network generation, the SIG model itself, forecast evaluation, and parameter transparency — are described too briefly for readers to properly assess or reproduce the work. The empirical validation is welcome but the heterogeneity of the underlying data across cities is not adequately handled. Substantial revision is required before this manuscript is suitable for publication.

Major Comments

1. Network generation is underdescribed and unvalidated

The network generation model is arguably the most novel methodological contribution of the paper, yet it receives less attention than almost any other component. The authors combine scale-free degree distributions, degree assortativity, role assortativity, spatial mixing, and a recurrent/one-time partner overlay into a single generative framework — but there is almost no validation that the realised networks actually exhibit the intended properties across the range of parameter combinations sampled.

The authors must demonstrate that realised networks behave as intended. Distributional summaries of key network properties — degree distribution shape and tail behaviour, assortativity coefficients, clustering — should be measured directly from the generated networks, not inferred from generative parameters. Cloud plots or scatter-plot matrices showing the joint distribution of realised network properties across the 100 MSM-like realisations would be highly informative and should be included. This is especially important because spatial mixing, scale-freeness, and multiple forms of assortativity are being combined simultaneously; their joint effects on network topology are non-trivial.

It is also unclear whether this generative model is novel or adapted from prior work. There are very few references in this section, and if the model is new it requires considerably more exposition and justification. Figure 1 should include quantitative network metrics (mean degree, clustering coefficient, assortativity coefficient) alongside the visual representations, which alone are insufficient to demonstrate that the three panels represent meaningfully different network structures.

2. Disconnected network components are not addressed

The authors do not address what happens when generated networks fragment into disconnected components, which is a well-known consequence of sparse or highly assortative networks. This is not a minor technical detail. The epidemic seeding strategy, the effective population size, and the interpretation of susceptible depletion all depend critically on whether transmission is constrained to the giant connected component or can propagate between components.

The authors should report the distribution of component sizes and the relative size of the largest connected component across realisations, and explicitly state whether epidemics are seeded within the giant component. Statistical summaries and plots of community/component size distributions should be added, particularly for the MSM-like networks where assortativity and spatial mixing parameters vary widely.

3. Unequal numbers of realisations across network classes are not justified

The study uses 30 homogeneous, 30 heterogeneous, and 100 MSM-like network realisations. No justification is provided for this asymmetry. Since primary comparisons are made across network types, unequal sample sizes introduce inconsistency in statistical power. The authors should either justify this design explicitly — for example via a power or precision argument — or adopt equal sample sizes throughout (e.g. 100 per class).

4. The SIG model, the paper’s central contribution, is poorly motivated

Equation 14 is presented without any mechanistic or intuitive explanation. Why a reverse-sigmoid? What biological or network process does this functional form represent? What does the floor parameter Rₛₗₒₒᵣ correspond to in terms of network dynamics — is it the reproduction number in the recurrent partner network once one-time contacts are exhausted? The authors gesture at this interpretation in the Discussion (lines 308–313) but it must appear in the Methods where the model is introduced.

The authors should also explore or at minimum discuss alternative functional forms for the attenuation. A reverse-sigmoid is one of many plausible choices; others include a generalised logistic, a Gompertz decay, or a piecewise linear approximation. The choice should be explicitly defended, as the paper’s primary claim rests on the superiority of this specific functional form.

5. Fitted versus fixed parameters are never distinguished

This is a significant transparency problem that runs throughout the paper. Table 3 lists parameters and priors by model, allowing the reader to infer which parameters are sampled. However, the paper never explicitly distinguishes between parameters that are fitted (estimated from data via HMC), parameters that are fixed from external sources, and parameters that are set by the simulation and therefore known when forecasting.

Specifically, the following are never clarified: (i) the generation time distribution ω(τ) — is this fixed from the literature or estimated? If fixed, from what source and with what values? (ii) population size N — in the empirical analysis this is unknown; is it fixed, estimated, or marginalised over? (iii) the incubation period in the empirical component — fixed or fitted? (iv) the initial number of infecteds I(0) — the prior is listed, but it is unclear whether this quantity is observable or latent in the real-world setting.

In the synthetic analysis the authors know the true network and transmission parameters because they generated the data. But in the empirical analysis, quantities like N and ω(τ) are unknown, and choices made here will materially affect R₀ estimates and therefore model comparison. This is entirely unaddressed. The authors must include a clear table distinguishing fixed from estimated parameters, presented separately for the synthetic and empirical analyses, with explicit justification for all fixed values.

6. The seven-model comparison is poorly justified

Several comparator models (EXPI and POWI in particular) perform so poorly — often by an order of magnitude relative to SIG — that their inclusion adds little analytical value and dilutes the key comparisons. The paper would benefit from a more principled selection: a pure statistical baseline (LRW), the classical renewal model (RRAND), and the most credible alternative attenuation approach (RBIAS). If all seven are retained, the authors must motivate why each is epidemiologically plausible in this setting. Currently the Forecasting Approaches section offers almost no justification for any model beyond a single reference, and the section is written exclusively for specialists with no background or intuition provided.

7. Day-of-week adjustment for New York data is insufficiently justified

The quasi-Poisson GLM approach to correcting weekend reporting artefacts in the NYC data is stated but not adequately motivated. It is not clear why this specific approach is appropriate, how large the estimated weekday effects are, or whether the adjustment meaningfully changes the shape of the epidemic curve. The authors should show before-and-after plots of the raw and adjusted NYC time series, and report the estimated weekday multipliers. The same level of scrutiny should be applied to the San Francisco data, which uses report date and may carry similar artefacts but receives no correction or comment.

8. Forecast evaluation choices are unmotivated

The choice to forecast 28 days ahead and evaluate only on days 22–28 is never explained. Why 28 days? Why evaluate only the final week of the horizon rather than performance across the full forecast window? Similarly, the choice of ten equally spaced forecast time points is stated without explanation of what the temporal gap between them is in absolute terms, or how this relates to the duration of individual epidemic curves. These are not minor implementation details — they materially influence which models appear to perform well, and they require justification.

Minor Comments

1. Line 161: “This includes one statistical models” — grammar error, should be “one statistical model.”

2. Equation 3 is presented as a free-floating display rather than being integrated into the preceding paragraph. It should be introduced inline (e.g. “...described by Equation 3 below”) and should end with a full stop or comma as appropriate to the surrounding sentence.

3. The caption of Table 2 should briefly define each of the seven model abbreviations rather than expecting readers to cross-reference back to the Methods.

4. Figure 2 requires considerably more explanation in its caption. Readers unfamiliar with CRPS decomposition will not understand what a bar dominated simultaneously by large red (overprediction) and green (underprediction) components indicates — namely a poorly calibrated model with high variance across replicates and insufficiently wide forecast intervals. One or two worked examples would substantially aid interpretation. The y-axis should also be labelled with units — it is not clear whether values represent individual case counts or some other scale, and without this context it is impossible to judge what constitutes a large or small error.

5. In Figure 5, the caption states that phases are shown in red, green, and blue, but these colours do not correspond to what is displayed in the figure. This must be corrected.

6. The figures appear to be placed at considerable distance from their corresponding discussion in the text, suggesting the Results section needs deeper integration of figures and narrative. The Results as currently written largely restates Table 2 numerically without sufficient interpretation.

7. The prior distributions in Table 3 (supplementary) are not discussed or defended in the main text. For a Bayesian model comparison, prior sensitivity is a legitimate concern and should at minimum be acknowledged.

8. Lines 205–206 state that CRPS reduces to mean absolute error for deterministic forecasts as a way of introducing the metric intuitively. However, all models in this paper produce probabilistic forecasts. This framing is therefore misleading and should be replaced with a more relevant intuitive description.

9. The paper acknowledges that one-time and recurrent contacts have different generation time distributions (uniform vs. geometric) but uses a single fixed generation time in all forecasting models. This is flagged as a future direction but is a current limitation that should be stated explicitly and prominently in the Limitations section.

10. It is generally not recommended to evaluate forecasts by fitting to cumulative incidence distributions. The authors should clarify whether this is what is intended by their CRPS evaluation setup and, if so, justify why.

11. The Forecasting Approaches section is written exclusively for specialists. It would benefit from at least one or two sentences of background and intuition before the equations, and from additional references motivating each modelling choice beyond the single citation currently provided.

Reviewer #2: Report on manuscript PCOMPBIOL-D-26-00282 “Forecasting of Infectious Disease Epidemics in Sexual Networks Using Attenuated Renewal Processes”

In their manuscript, the authors report on an agent-based model to study the spread of an infectious disease. They consider three different networks and mathematically describe the spreading process by an SEIR model. The networks considered are homogeneous networks (uniform degree distribution), heterogeneous networks (truncated power-law degree distribution), and networks designed to close capture reality. The spreading of a sexually transmitted disease among a population of men who have sex with men is considered.

My main concern is that the outline of the study (modeling and network generation/relevance) is not clear.

At first, I got the impression that the authors consider a stochastic agent-based model. Then, however, there is also a statement on a log-random walk and a renewal model. Overall, the section “Forecasting approaches” and Eqs.(7)-(15) do not provide sufficient information to understand the model setup. It also unclear how the probability given in Eq.(1) is normalized. Furthermore, why is SEIR a suitable model?

There are similar concerns about the networks considered:

1. The homogeneous network is certainly not realistic. Besides, it is unclear why a uniform degree distribution leads to a homogeneous network. Do the authors mean a regular network where every node has the same degree?

2. Table 1 is not informative as it omits information on every network type.

3. Table 1 provides ranges for many parameters. Which were used in the end? Did the authors consider a systematic parameter scan?

4. The truncated power-law is also confusing. How is a range of power-law exponents between -2 and -1.6 justified for a contact network?

5. The idea of spatial proximity does not agree with a network modeling approach. The intention for a spatial embedding is unclear.

6. Shouldn’t the underlying network have a temporal component?

Conceptionally, the research strategy remains unclear. If a real-world case is available, the authors could compare their models to the ground truth.

For the “Fitting appraoch”, the authors refer to a number of methods without introducing them properly.

Without providing results of the simulation (time series, parameter scans etc.), the study is not reproducible.

I appreciate that the code and data were made available via github. The UK data, however, is not included and can only be accessed via a separate data request.

To sum up, there are too many open questions about the design of the study. It could very well be that it is simply a presentation issue. In the present form, however, the manuscript is not understandable and makes it impossible to follow the reasoning of the authors. A complete revision would be necessary that would turn the resubmisson in an entirely new manuscript. Therefore, I recommend to rejection.

**********

**Have the authors made all data and (if applicable) computational code underlying the findings in their manuscript fully available?**

The PLOS Data policy requires authors to make all data and code underlying the findings described in their manuscript fully available without restriction, with rare exception (please refer to the Data Availability Statement in the manuscript PDF file). The data and code should be provided as part of the manuscript or its supporting information, or deposited to a public repository. For example, in addition to summary statistics, the data points behind means, medians and variance measures should be available. If there are restrictions on publicly sharing data or code —e.g. participant privacy or use of data from a third party—those must be specified.

Reviewer #1: None

Reviewer #2: **No:**  I appreciate that the code and data were made available via github. The UK data, however, is not included and can only be accessed via a separate data request.

**********

PLOS authors have the option to publish the peer review history of their article (what does this mean?). If published, this will include your full peer review and any attached files.

If you choose “no”, your identity will remain anonymous but your review may still be made public.

**Do you want your identity to be public for this peer review?**  For information about this choice, including consent withdrawal, please see our Privacy Policy.

Reviewer #1: No

Reviewer #2: No

[NOTE: If reviewer comments were submitted as an attachment file, they will be attached to this email and accessible via the submission site. Please log into your account, locate the manuscript record, and check for the action link "View Attachments". If this link does not appear, there are no attachment files.]

**Figure resubmission:**

**Reproducibility:**

?>

# Author comment: Author response to Decision Letter 1

**DOI:** 10.1371/journal.pcbi.1013987.r002

12 Aug 2026

Attachment Submitted filename: response_to_reviewers_vF.pdf

# Peer review: Decision Letter 1

**DOI:** 10.1371/journal.pcbi.1013987.r003

**Authors:** Jennifer Flegg, Konstantin B. Blyuss

17 Sep 2026

Dear Dr Green,

We are pleased to inform you that your manuscript 'Forecasting of Infectious Disease Epidemics in Sexual Networks Using Attenuated Renewal Processes' has been provisionally accepted for publication in PLOS Computational Biology.

Before your manuscript can be formally accepted you will need to complete some formatting changes, which you will receive in a follow up email. A member of our team will be in touch with a set of requests.

Please note that your manuscript will not be scheduled for publication until you have made the required changes, so a swift response is appreciated.

IMPORTANT: The editorial review process is now complete. PLOS will only permit corrections to spelling, formatting or significant scientific errors from this point onwards. Requests for major changes, or any which affect the scientific understanding of your work, will cause delays to the publication date of your manuscript.

Should you, your institution's press office or the journal office choose to press release your paper, you will automatically be opted out of early publication. We ask that you notify us now if you or your institution is planning to press release the article. All press must be co-ordinated with PLOS.

Thank you again for supporting Open Access publishing; we are looking forward to publishing your work in PLOS Computational Biology.

Best regards,

Konstantin B. Blyuss

Academic Editor

PLOS Computational Biology

Jennifer Flegg

Section Editor

PLOS Computational Biology

***********************************************************

Reviewer's Responses to Questions

**Comments to the Authors:**

**Please note here if the review is uploaded as an attachment.**

Reviewer #1: I thank the authors for engaging with and addressing my comments. I have no further comments and I am happy to recommend the paper for publication.

Reviewer #2: Report on the revised manuscript PCOMPBIOL-D-26-00282R1 “Forecasting of Infectious Disease Epidemics in Sexual Networks Using Attenuated Renewal Processes”

In their revision, the authors substantially improved (and – in parts – corrected) the manuscript. I very much appreciate the additional schematic figure (new Fig.1) that removes the main concern in my earlier report. The workflow is now much more accessible.

Furthermore, the authors adequately responded to my criticism and performed carefully changes and extensions to the text. Among others, these include a better justification of the model setup (comments 1 and 3), a precise definition of the “homogeneous network” and an increased clarity on the parameters used (comments 5 and 6) and network-data handling resulting. This led to updated results presented in Table 2, Figs. 3, 4, and 6.

All sections have been revised. The discussion of the results has been extended (partly in longer figure captions), and the Supplementary Material now contains further information on the study and network validation. For the latter, the Spearman correlation coefficients (Supplementary Figure 1) are hard to read, though. Maybe, a different presentation mode (black numbers on color-shaded squares) would help. Finally, the references in the Supplementary Material (Table 1) should be added in that document.

To sum up, I congratulate the authors on their thorough, general overhaul of the manuscript, which has improved in many ways. Now, I can better follow their line of reasoning and see the merit of their work. In my opinion, the manuscript can be accepted for publication with the small changes to the Supplementary Material mentioned above.

**********

**Have the authors made all data and (if applicable) computational code underlying the findings in their manuscript fully available?**

The PLOS Data policy requires authors to make all data and code underlying the findings described in their manuscript fully available without restriction, with rare exception (please refer to the Data Availability Statement in the manuscript PDF file). The data and code should be provided as part of the manuscript or its supporting information, or deposited to a public repository. For example, in addition to summary statistics, the data points behind means, medians and variance measures should be available. If there are restrictions on publicly sharing data or code —e.g. participant privacy or use of data from a third party—those must be specified.

Reviewer #1: None

Reviewer #2: Yes

**********

PLOS authors have the option to publish the peer review history of their article (what does this mean?). If published, this will include your full peer review and any attached files.

If you choose “no”, your identity will remain anonymous but your review may still be made public.

**Do you want your identity to be public for this peer review?**  For information about this choice, including consent withdrawal, please see our Privacy Policy.

Reviewer #1: No

Reviewer #2: No

# Editor report: Acceptance letter

**DOI:** 10.1371/journal.pcbi.1013987.r004

**Authors:** Jennifer Flegg, Konstantin B. Blyuss

PCOMPBIOL-D-26-00282R1

Forecasting of Infectious Disease Epidemics in Sexual Networks Using Attenuated Renewal Processes

Dear Dr Green,

I am pleased to inform you that your manuscript has been formally accepted for publication in PLOS Computational Biology. Your manuscript is now with our production department and you will be notified of the publication date in due course.

The corresponding author will soon be receiving a typeset proof for review, to ensure errors have not been introduced during production. Please review the PDF proof of your manuscript carefully, as this is the last chance to correct any errors. Please note that major changes, or those which affect the scientific understanding of the work, will likely cause delays to the publication date of your manuscript.

Soon after your final files are uploaded, unless you have opted out, the early version of your manuscript will be published online. The date of the early version will be your article's publication date. The final article will be published to the same URL, and all versions of the paper will be accessible to readers.

For Research, Software, and Methods articles, you will receive an invoice from PLOS for your publication fee after your manuscript has reached the completed accept phase. If you receive an email requesting payment before acceptance or for any other service, this may be a phishing scheme. Learn how to identify phishing emails and protect your accounts at https://explore.plos.org/phishing.

Thank you again for supporting PLOS Computational Biology and open-access publishing. We are looking forward to publishing your work!

With kind regards,

Sharmila Kamatchi

PLOS Computational Biology | Carlyle House, Carlyle Road, Cambridge CB4 3DN | United Kingdom ploscompbiol@plos.org | Phone +44 (0) 1223-442824 | ploscompbiol.org | @PLOSCompBiol
